# Systematic engineering pinpoints a versatile strategy for the expression of functional cytochrome P450 enzymes in *Escherichia coli* cell factories

**DOI:** 10.1186/s12934-023-02219-7

**Published:** 2023-10-25

**Authors:** Michal Poborsky, Christoph Crocoll, Mohammed Saddik Motawie, Barbara Ann Halkier

**Affiliations:** 1 Department of Plant and Environmental Sciences, DynaMo Center of Excellence, University of Copenhagen, Thorvaldsensvej 40, Frederiksberg C, 1871 Denmark; 2 Department of Plant and Environmental Sciences, Section for Plant Biochemistry, University of Copenhagen, Thorvaldsensvej 40, Frederiksberg C, 1871 Denmark

## Abstract

Production of plant secondary metabolites in engineered microorganisms provides a scalable and sustainable alternative to their sourcing from nature or through chemical synthesis. However, the biosynthesis of many valuable plant-derived products relies on cytochromes P450 – enzymes notoriously difficult to express in microbes. To improve their expression in *Escherichia coli*, an arsenal of engineering strategies was developed, often paired with an extensive screening of enzyme variants. Here, attempting to identify a broadly applicable strategy, we systematically evaluated six common cytochrome P450 N-terminal modifications and their effect on in vivo activity of enzymes from the CYP79 and CYP83 families. We found that transmembrane domain truncation was the only modification with a significantly positive effect for all seven tested enzymes, increasing their product titres by 2- to 170-fold. Furthermore, when comparing the changes in the protein titre and product generation, we show that higher protein expression does not directly translate to higher in vivo activity, thus making the protein titre an unreliable screening target in the context of cell factories. We propose the transmembrane domain truncation as a first-line approach that enables the expression of wide range of highly active P450 enzymes in *E. coli* and circumvents the time-consuming screening process. Our results challenge the notion that the engineering strategy must be tailored for each individual cytochrome P450 enzyme and have the potential to simplify and accelerate the future design of *E. coli* cell factories.

**Supplementary Information:**

The online version contains supplementary material available at 10.1186/s12934-023-02219-7.

## Preprint severs

Biorxiv, CC-BY-NC-ND 4.0 International license. 10.1101/2022.12.13.520134v1.abstract.

## Introduction

Cytochromes P450 (CYPs or P450s) are amongst the most versatile enzymes in nature and draw the attention of metabolic engineers with their ability to catalyse unique reactions on a myriad of complex substrates. *In planta*, P450s unlock the diversity of secondary metabolism [1] and play a key role in the biosynthesis of natural products used as medicines, cosmetics, colourants, and flavours [2, 3]. Reconstituting these biosynthetic pathways in microbial cell factories, such as *Escherichia coli* or *Saccharomyces cerevisiae*, allows the production of high-value natural products without the problems associated with the native hosts: seasonal nature of the production, low amounts of the product per plant, and tedious extraction from plant material. Well-known examples include the semi-synthetic production of the antimalarial drug artemisinin [4]; biosynthesis of taxadiene, an intermediate of the anti-cancer medicine Taxol [5]; and *de novo* biosynthesis of analgesic opioids [6, 7]. Still, functional expression of heterologous P450s remains a challenge that researchers must address to reach competitive production levels of plant pathways in microbes.

All plant P450s require a redox partner for electron transfer and most localise on the membrane of the endoplasmic reticulum [8, 9], making *E. coli* – without eukaryotic organelles and no native P450 enzymes – a particularly challenging host. The N-terminal hydrophobic amino acids, which anchor P450s in membranes and act as a membrane localisation signal, cause the proteins to aggregate in inclusion bodies if the proteins cannot assume their native fold at a pace matching the speed of translation [10, 11]. Larson et al. pioneered the strategy of N-terminal truncation, removing the hydrophobic amino acids of rabbit CYP2E1 and showing that the enzyme maintains its original activity [12]. Barnes et al. optimised the 5’ codons of bovine CYP17A, increasing the translation initiation speed and enabling the protein to express in *E. coli* [13]. N-terminal truncation and insertion of the MALLLAVF peptide, the so-called Barnes sequence, were later used to establish the expression of many other eukaryotic P450s in bacteria [14–18]. Because these modifications can increase the ratio of P450s localised in the cytosol [19, 20], alternative techniques were developed to maintain P450 membrane localisation: insertion of leader sequences from bacterial membrane proteins [21] and exchange of the N-terminal domain with transmembrane sequences from *E. coli* membrane proteins [22, 23] or well-expressed eukaryotic P450 enzymes [24].

Although researchers can now choose from an arsenal of engineering strategies, no clear favourite exists and establishing novel plant pathways requires extensive optimisation and screening of the P450 enzyme expression and functionality [22, 23]. Considering that most of the available research has focused on increasing the P450 protein titre, which does not always translate to higher in vivo activity [25], it is difficult to use previous results to guide the P450 engineering strategy in the context of cell factories. Furthermore, plant biosynthetic pathways often rely on multiple P450-mediated steps [1], while most studies in the field aim to overexpress only a single P450 enzyme at a time [26].

**Fig. 1:**
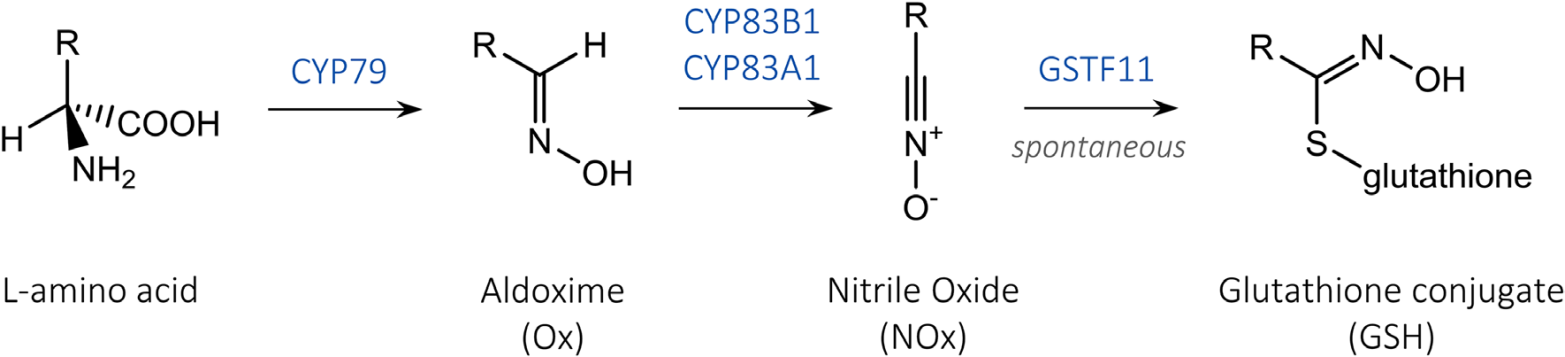
The role of CYP79 and CYP83 in glucosinolate pathway. The first two steps of glucosinolate biosynthesis are catalysed by P450 enzymes from CYP79 and CYP83 families that convert amino acids into oximes and further into nitrile oxides, which can be conjugated with glutathione by a glutathione-S-transferase (GST) or spontaneously. We measured the generation of phenylalanine-derived products to evaluate the effect of N-terminal sequence modifications on P450 functionality in *E. coli*: phenylacetaldoxime (Phe-Ox), when engineering CYP79A2 alone, and *S-*phenylacetohydroxymoyl-L-glutathione (Phe-GSH), when engineering CYP79A2 together with CYP83s

Here, we suggest a versatile strategy to express highly functional P450s in *E. coli* cell factories by systematically comparing six common N-terminal modifications. We evaluate the changes of in vivo activity of CYP79A2 together with two members of the CYP83 family and then validate the best strategy on four additional P450 enzymes. CYP79 and CYP83 catalyse two consecutive steps at the entrance to the glucosinolate pathway and accept standard amino acids as substrates (Fig. 1) [27]. This makes them a convenient model system for screening of product generation that avoids the need to feed complex substrates typical for plant secondary metabolism and allows the evaluation of concurrent engineering of multiple P450s.

## Results

### N-terminal sequence modifications affect CYP79A2 functionality and expression levels

**Fig. 2:**
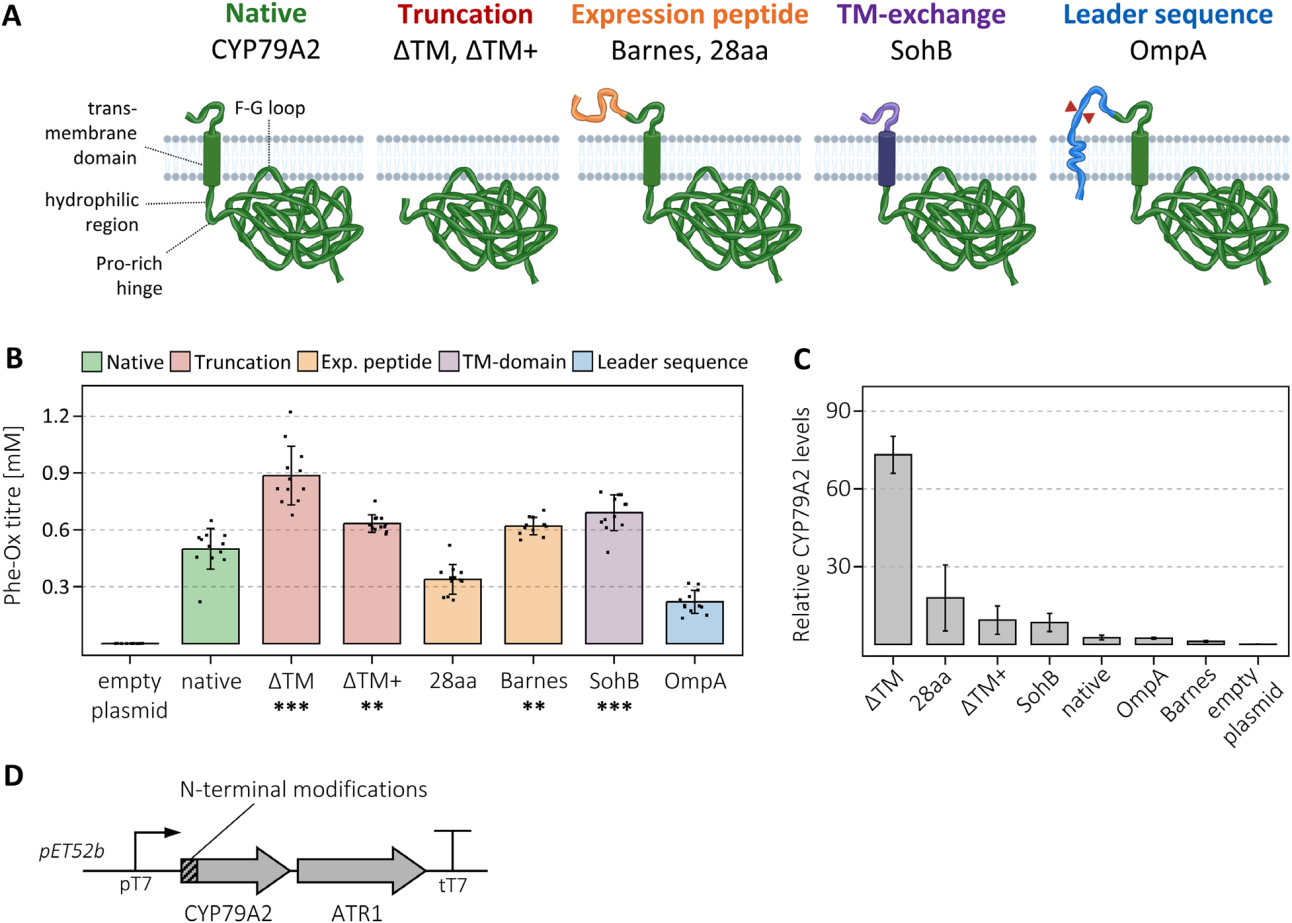
Effect of CYP79A2 N-terminal modifications on the production of phenylacetaldoxime (Phe-Ox). **A** N-terminal sequence modifications used to improve function of P450 enzymes include: (1) truncation of the transmembrane domain (ΔTM) or both transmembrane domain with adjacent hydrophilic region (ΔTM+), (2) expression enhancing peptides MALLLAVF (Barnes) or 28 amino acid tag (28aa), (3) transmembrane domain exchange with *E. coli* native probable protease (SohB), and (4) insertion of leader sequence from outer membrane protein A (OmpA) in front of the full-length protein. **B** Phe-Ox titres produced from phenylalanine by native and engineered variants of CYP79A2. All strains were grown in 12 biological replicates across two independent experiments. Error bars represent standard deviation from the mean and outliers were removed from the data. Student’s upper-tailed *t* test denotes a significant increase in Phe-Ox titre compared to native with *p* value (with Bonferroni adjustment), ** *p* < 0.01, *** *p* < 0.001. Exact levels of Phe-Ox, Phe and OD600 are listed in Table S1. **C** Relative quantification of CYP79A2 levels in strains expressing the native and engineered variants of CYP79A2, showing a representative peptide of CYP79A2 normalized to expression of *E. coli* isocitrate dehydrogenase. The bars represent the mean of 3–5 biological replicates from one of the experiments in B and error bars represent standard deviation from the mean (Table S2). Additional CYP79A2 peptides can be found in Fig. S1. **D** Illustration of the CYP79A2-ATR1 operons as cloned into pET52b vector. Full-length cytochrome P450 oxidoreductase (ATR1) was co-expressed to supply electrons for the regeneration of P450 enzymes

We chose the conversion of phenylalanine to phenylacetaldoxime (Phe-Ox) catalysed by CYP79A2 for the initial assessment of N-terminal modifications because CYP79A2 constitutes a bottleneck in its biosynthetic pathways [27, 28], and it could be improved by N-terminal truncation [29] and the introduction of Barnes sequence [28]. We cloned the full-length, codon-optimized CYP79A2 from *Arabidopsis thaliana* and six engineered variants with different N-terminal modifications into operons with *A. thaliana* reductase 1 (ATR1, Fig. 2D). All tested modifications were previously shown to increase the functional expression of P450s and fall into four general categories: (1) N-terminal truncation by removing either the transmembrane domain (ΔTM) or both the transmembrane domain and the adjacent hydrophilic region (ΔTM+) [12, 29], (2) 5’-codon optimisation by inserting short expression enhancing peptides in front of the P450 protein sequence (Barnes and 28aa), (3) transmembrane domain exchange with *E. coli* native membrane anchor from SohB probable protease (SohB) [22], and (4) introduction of *E. coli* leader sequence from outer membrane protein A (OmpA) [21] (Fig. 2A).

We assayed CYP79A2 variants by measuring the generation of Phe-Ox after 72 h fermentation in T7 autoinducing media (Fig. 2B). Although CYP79A2 was functional without any sequence modifications, producing 0.52 mM Phe-Ox, many engineered variants reached significantly higher titres. Those with the transmembrane domain removed or replaced were the top producers and [ΔTM]CYP79A2, where we truncated the transmembrane domain, but left the hydrophilic region intact, reached the highest Phe-Ox titre at 0.89 mM. The introduction of the Barnes sequence was beneficial only when the MALLLAVF peptide was inserted in front of the full-length enzyme, rather than substituting the first eight amino acids (Fig. S2). Using OmpA leader sequence or 28aa tag impaired CYP79A2 function, reducing Phe-Ox titre by approximately 30%. Because ATR2 and Δ44ATR2 are often used in literature, we compared the different reductases and their effect on [ΔTM]CYP79A2 activity. In our expression system, ATR1 was superior to both and led to 2-fold and 3-fold higher Phe-Ox titres than ATR2 and Δ44ATR2, respectively (Fig. S3) and was therefore used in all following experiments. Ensuring the rich medium did not skew our results, we have compared the strains expressing [ΔTM]CYP79A2 and native CYP79A2 in supplemented M9 minimal media and obtained comparable improvement as in TB medium (Fig. S4).

To relate the metabolite data with the P450 protein titre, we performed targeted proteomics measuring CYP79A2 protein expression levels on six of the replicates used above (Fig. 2C). We observed a disparity between the effect the modifications had on CYP79A2 protein expression compared to the increase in product generation. [ΔTM]CYP79A2 showed the highest protein levels and highest Phe-Ox production, but the relative protein titre increased by 28-fold, while Phe-Ox titre improved by only 1.8-fold. The introduction of 28aa tag increase protein levels 7-fold compared to native enzyme, while decreasing Phe-Ox production by 40%. These results corroborate previous reports that protein titre is not indicative of in vivo activity of P450 enzymes [25]. In the following sections, we continue with [ΔTM]CYP79A2 as the best-performing variant using product generation as the metric to evaluate our engineering strategies.

### Concurrent engineering of a dual P450 module

**Fig. 3:**
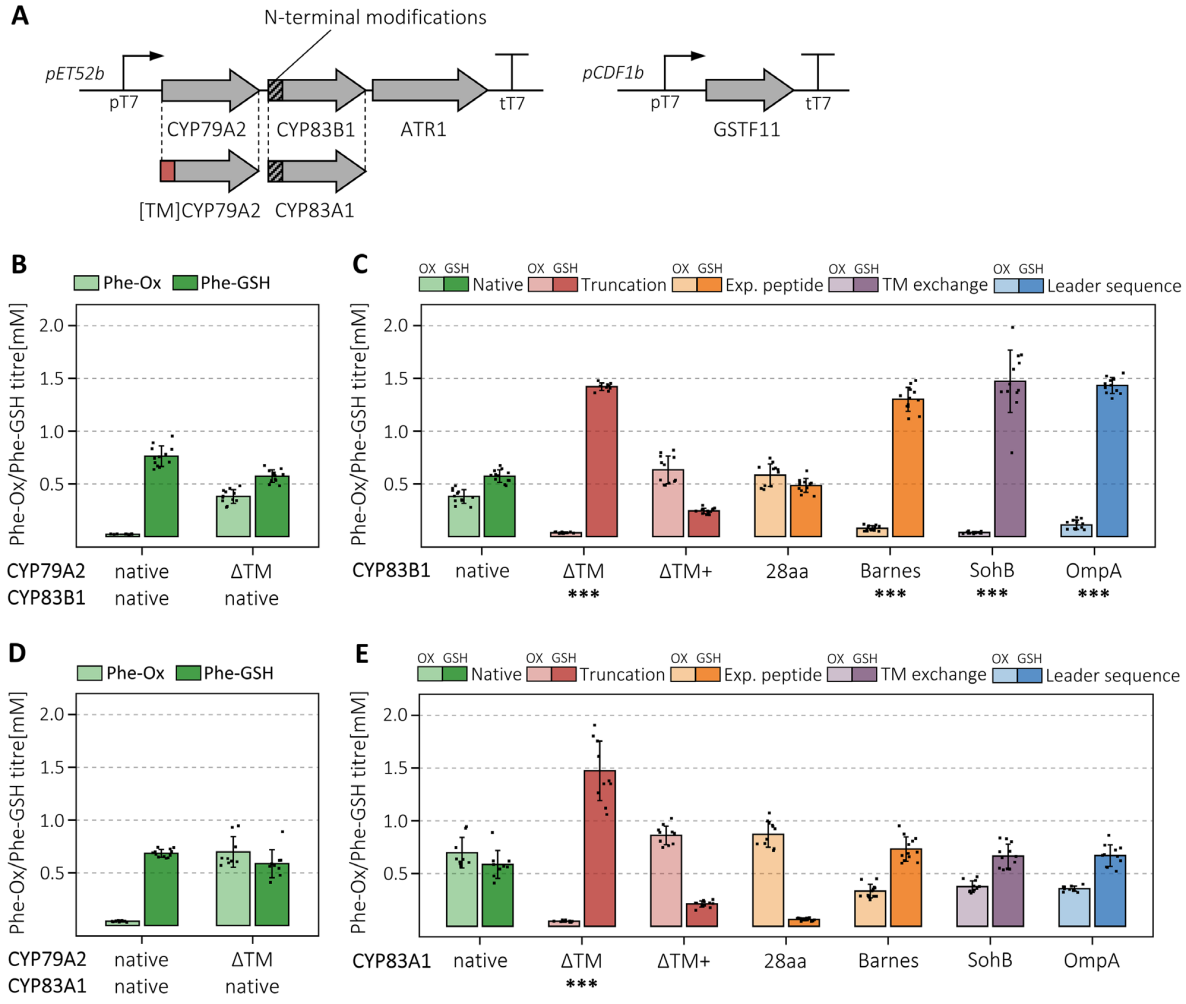
Engineering of the dual P450 module of CYP79A2 with CYP83B1 or CYP83A1. **A** The design of the expression constructs to examine the combinatorial effect of engineering the two P450s together. We paired the native and [ΔTM]-CYP79A2 with variants of the two CYP83s and co-expressed the P450 oxidoreductase ATR1 and glutathione transferase F11. **B, D** Production of phenylacetaldoxime (Phe-Ox, light colours) and *S-*phenylacetohydroxymoyl-L-glutathione (Phe-GSH, dark colours) by native and [ΔTM]CYP79A2 with native CYP83B1 and CYP83A1, respectively. **C, E** Production of Phe-Ox and Phe-GSH by [ΔTM]CYP79A2 with native and modified variants of CYP83B1 and CYP83A1, respectively. Bars are colour coded by the type of modification applied to CYP83 enzymes. All strains were grown in 12 biological replicates across two independent experiments. Error bars represent standard deviation from the mean and outliers were removed from the data. For **C** and **E**, Student’s upper-tailed *t* test denotes a significant increase in Phe-GSH titre compared to [ΔTM]CYP79A2 and native CYP83s with *p* value (with Holm adjustment), *** *p* < 0.001. Exact levels of Phe-Ox, Phe-GSH, Phe and OD600 are listed in Table S3

Plant biosynthetic pathways for valuable secondary metabolites often proceed through multiple P450-mediated steps [1]. To study the simultaneous engineering of two P450 enzymes, we paired native and truncated CYP79A2 with variants of CYP83B1 or CYP83A1, which both convert Phe-Ox into phenylacetonitrile oxide (Fig. 1). We expressed CYP79A2 with either one of the CYP83s and ATR1 from a single operon under the control of T7 promoter (Fig. 3A). When assaying the dual P450 module, we measured the generation of *S-*phenylacetohydroxymoyl-L-glutathione (Phe-GSH, Fig. 1), a more suitable product to quantify than the reactive and unstable nitrile oxide [30]. Although the conjugation of nitrile oxides with glutathione can occur spontaneously, we co-expressed glutathione-S-transferase F11 (GSTF11) alongside the P450s and ATR1 to improve the formation of Phe-GSH (Fig. 3A) [31].

We observed that strains with native CYP83s failed to convert the increased supply of Phe-Ox by [ΔTM]CYP79A2 into nitrile oxide, producing less Phe-GSH than with native CYP79A2 and accumulating Phe-Ox instead (Fig. 3B and D). This was not specific to the truncated CYP79A2, as expressing the SohB variant led to the same result (Fig. S5). To achieve nearly complete conversion, we had to engineer the N-termini of both CYP79A2 and CYP83s. Although the highest Phe-GSH titres were similar between the CYP83s, interestingly, the effect of the N-terminal modifications varied between the two enzymes. For CYP83B1, multiple variants showed a significant increase in product titre with ΔTM, SohB and OmpA variants generating approximately 1.5 mM Phe-GSH, more than double compared to the native control (Fig. 3C). For CYP83A1, only ΔTM variant had a significantly positive effect on product titre and generated 1.47 mM Phe-GSH, on par with CYP83B1 (Fig. 3E). Our results suggest ΔTM N-terminal truncation is superior to the other approaches as it was the only modification that significantly increased the product generation by all three enzymes CYP79A2, CYP83B1 and CYP83A1.

### Transmembrane domain truncation as a first-line approach for P450 functional expression in *E. coli*

To validate transmembrane domain truncation as a strategy that could be applied to a broad range of P450s, we chose four additional CYP79 enzymes with diverse N-terminal sequences, distinct substrate preferences and different plant origins. CYP79A1 from *Sorghum bicolor* converts tyrosine into its corresponding oxime [32], CYP79B2 from *A. thaliana* accepts tryptophan as the sole substrate [33], CYP79D2 from *Manihot esculenta* acts on both valine and isoleucine [34], and CYP79F6 from *Barbarea vulgaris* accepts only non-canonical, carbon chain-elongated amino acids (here homophenylalanine) [35]. We cloned the four CYP79s, CYP83 and ATR1 into operons where both CYP79 and CYP83 were either native full-length or with the transmembrane domain truncated.

**Fig. 4:**
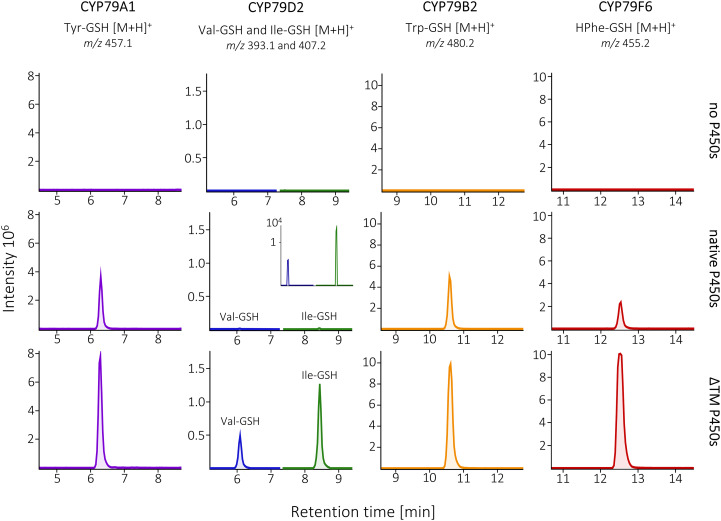
Validating the broad applicability of the transmembrane domain truncation (ΔTM) on four additional CYP79 enzymes with distinct substrate specificities and different plant origin. We expressed native and ΔTM variants of *Sb*CYP79A1 (*Sorghum bicolor*, tyrosine-specific), *Me*CYP79D2 (*Manihot esculenta*, valine- and isoleucine-specific), *At*CYP79B2 (*Arabidopsis thaliana*, tryptophan-specific) and *Bv*CYP79F6 (*Barbarea vulgaris*, homophenylalanine (HPhe)-specific), together with CYP83s, ATR1 and GSTF11. Native and truncated CYP83B1 was paired with *Sb*CYP79A1, *Me*CYP79D2 and *At*CYP79B2, whereas CYP83A1 was co-expressed with *Bv*CYP79F6. We measured the generation of putative glutathione conjugates derived from the respective amino acids by Q-TOF LC-MS/MS. The presented extracted ion chromatograms are representative of 4 biological replicates and the characteristic fragmentation patters for each glutathione conjugate are shown in Fig S6

We assayed the enzymes by measuring the generation of putative glutathione conjugates derived from their respective amino acid substrates by tandem mass spectrometry (Q-TOF LC-MS/MS), comparing the relative production in strains expressing the native and truncated P450s (Fig. 4). We identified the glutathione conjugates by extracting molecular features with matching *m/z* ratios and a characteristic fragmentation pattern in the mass spectrum [36, 37], which we confirmed with Phe-GSH standard (Fig. S6). The results further corroborated the broad applicability of ΔTM as the transmembrane domain truncation led to higher product accumulation for all four new CYP79s. Although CYP79A1 and CYP79B2 were functional without any modification, truncating the enzymes yielded almost double of their respective products despite being limited by insufficient supply of tyrosine and tryptophan in TB media (Fig. S7). The strain with [ΔTM]CYP79F6 and [ΔTM]CYP83A1 produced almost 7-fold more homo-phenylalanine-derived glutathione conjugate than the strain with native P450s and in the case of CYP79D2, which showed only trace activity without truncation, the improvement was 150-fold and 170-fold for valine and isoleucine products, respectively. Taken together, we show that transmembrane domain truncation improves functional expression and in vivo product accumulation for all seven studied P450 enzymes, being especially effective for enzymes with low native activity.

## Discussion

We produced a broad and systematic screening of six common P450 N-terminal sequence modifications evaluating their effect on the in vivo enzymatic activity. Using two consecutive steps catalysed by CYP79A2 and either CYP83A1 or CYP83B1 as a model system, we found that truncation of the transmembrane domain outperformed the other N-terminal modifications as it significantly improved product generation across all three P450 enzymes. We sought to validate this finding by truncating four additional CYP79 enzymes and showed that ΔTM variants again led to increased product titres in all four cases. Amongst the six modifications, only the transmembrane domain truncation was beneficial for all studied combinations of enzymes from CYP79 and CYP83 families, improving the product titres 2- to 170-fold. This strongly suggests that the transmembrane domain truncation serves as a versatile strategy to improve the functional expression of plant P450s in *E. coli*. Moreover, in the context of microbial cell factories, we demonstrated that in pathways with multiple P450s concentrating on the rate-limiting step is insufficient and all P450s must be engineered concurrently.

Classically, P450 engineering studies measure protein titre as a metric of success [25, 26]. However, we observed a disparity between the effect the modifications had on the protein levels and the in vivo activity of CYP79A2 enzyme. ΔTM, ΔTM + and SohB variants improved protein expression far greater than product titre whereas [28aa]CYP79A2 increased protein levels 7-fold while reducing Phe-Ox production by 30% compared to the native enzyme. Zhou et al. reviewed several studies that report a similar discrepancy [25] and Christensen et al. showed that CYP79A1 fused to a membrane anchor of *E. coli* signal peptidase 1 was amongst the variants with the highest protein levels but only marginally improved in vivo activity [22]. Changes at the N-terminus can increase the P450 fraction in the cytosol [9, 19, 20, 26], limiting their access to the P450 reductase, and may interfere with the protein-protein interaction between the reductase and the P450s on the membrane [38]. It is possible that the modifications impair the electron transfer between the reductase and the P450 variants, where protein amount does not translate to in vivo activity. Furthermore, at the very high expression levels of [ΔTM]CYP79A2, the availability of substrates, heme or NADPH may replace protein titre as the limiting factor. Together, these results indicate that screening P450 protein expression levels is insufficient to guide the engineering strategy in the context of cell factories. While screening P450 in vivo activity is more exact, it becomes cumbersome at scale requiring access to diverse and complex substrates as well as established quantification methods for the generated products.

We set out to identify an engineering strategy that could be applied as a first-line approach without screening a library of variants, as, in our opinion, no clear favourite has emerged in literature. Available studies use different growth conditions and combine multiple modifications to tailor individual P450 enzymes for the highest possible protein titre, making it difficult to abstract a broadly applicable engineering strategy. We studied the modifications individually and found that only ΔTM caused a significant increase in product generation across all tested enzymes, and while other modifications could lead to similar improvements, they failed to have a consistently positive effect. Barnes and SohB variants were significantly better than native CYP79A2 and CYP83B1 but caused no statistically significant change in CYP83A1. OmpA was among the top variants for CYP83B1 but led to a dramatic decrease in Phe-Ox production by CYP79A2. The final culture density was not a general determinant of high in vivo activity, but specifically for OmpA and Barnes variants with low product titres, slower growth may contribute to their poor performance (Table S1 and S3). These results show that the transmembrane domain is a major factor that impedes the functional expression of P450s in *E. coli*, and its truncation, in front of the hydrophilic region, is sufficient to alleviate the problem in a wide range of P450 enzymes.

Balancing the expression of individual enzymes or modules is a standard approach in pathway engineering. We noticed a similar requirement for P450 engineering in pathways containing multiple P450 enzymes. [ΔTM]CYP79A2, which catalyses the rate-limiting reaction at the entry point of the glucosinolate pathway [27, 28], produced almost double Phe-Ox over the native enzyme, but this benefit was lost upon adding the subsequent unmodified CYP83s, which were unable to convert the extra Phe-Ox into nitrile oxide. Only when engineering both CYP79A2 and CYP83s, we successfully translated the doubled Phe-Ox titre into doubled Phe-GSH. As P450s draw from shared cellular resources such as heme incorporation, electron supply through NADPH, and electron transfer from NADPH to P450 by cytochrome P450 reductase, the 28-fold increase in expression we observed for [ΔTM]CYP79A2 might cause CYP79A2 to monopolize the access to these resources and, in turn, impair CYP83 activity.

Although optimization and validation at a larger scale is necessary, we expect our findings to translate well from a 24-well plate screening to a bioreactor setting. Bioreactors provide a more controlled fermentation environment, which can support much higher bacterial biomass and thus higher product titre. Especially the control over dissolved oxygen and aeration rate will have a great impact on the in vivo activity of P450s, which require oxygen for catalysis. Furthermore, due to cost, minimal media is common for fermentation at scale and in the industry. We have performed a control experiment in supplemented M9 minimal media showing that the benefit of N-terminal truncation is independent of rich media and high product titres are maintained (Fig. S4). Taken together, we identify the N-terminal truncation as the best and the most versatile modification to achieve high in vivo activity across a wide range of plant P450 enzymes in *E. coli.* Our results emphasise the discrepancy between P450 protein titre and in vivo activity, suggesting that screening of product generation is necessary to guide P450 engineering. To avoid the extensive screening of various N-terminal modifications, we propose ΔTM as a first-line approach to yield highly functional P450 enzymes and aid future design and establishment of P450-containing biosynthetic pathways in *E. coli* cell factories.

## Materials and methods

**Oligonucleotides, Plasmids, Bacterial strains, Transformation protocol and LC-MS/MS procedures for small molecules and peptides.** Detailed description can be found in Supplementary Material.

### Construction of operons for P450 expression

Purified PCR products were assembled using an adapted USER protocol from Geu-Flores et al. [39] and Cavaleiro et al. [40] in a 10 µL reaction as follows: 1 µL USER enzyme mix, 1 µL 10x CutSmart buffer, 1 unit of DpnI, 20 ng PCR product of the plasmid backbone and 40 ng PCR product of all fragments to assemble. The reaction was incubated for 1 h at 37 °C, then at gradually decreasing temperatures around the melting temperature (T_m_) of USER overhangs (31 − 26 °C) with 5 min at each temperature step and finished with 30 min at 10 °C. The heterologous genes were assembled into synthetic operons with a maximum of three genes per operon. All operons were flanked by consensus T7 promoters with T7 terminators, and all genes shared the same ribosome binding site originating from the T7 bacteriophage major capsid protein.

**Table 1:** Seven P450 enzymes of the glucosinolate pathway from four different plants were targeted for engineering in our study. CYP79s control the amino acid substrate entering the pathway and we selected five with distinct substrate preferences and plant origin. The subsequent CYP83s are promiscuous and convert any oximes generated by CYP79s. ΔTM and ΔTM + represent the residues that were truncated after the membrane anchor or after the membrane anchor plus the hydrophilic region. Complete amino acid sequences of the enzymes can be found in Table S5

Enzyme	Substrates	ΔTM	ΔTM+	Plant origin
CYP79A1	Tyr	Δ38		*Sorghum bicolor*
CYP79A2	Phe	Δ14	Δ23	*Arabidopsis thaliana*
CYP79B2	Trp	Δ42		*Arabidopsis thaliana*
CYP79D2	Val, Ile	Δ41		*Manihot esculenta*
CYP79F6	HPhe	Δ29		*Barbarea Vulgaris*
CYP83A1	AA oximes	Δ23	Δ28	*Arabidopsis thaliana*
CYP83B1	AA oximes	Δ21	Δ29	*Arabidopsis thaliana*

To define transmembrane domains of P450 enzymes, we used Uniprot annotations in combination with a transmembrane domain prediction tool TMHMM v2.0 (now deprecated in favour of DeepTHMHH) [41] (Table 1). To identify an appropriate truncation site for ΔTM+, the hydrophilic region was annotated as a short amino acid stretch abundant in arginine and lysine residues together with other hydrophilic amino acids. The P450 N-terminal sequence modifications include: (1) truncation of the membrane domain in front of the hydrophilic region (ΔTM), (2) truncation of the membrane domain after the hydrophilic region (ΔTM+), (3) addition of Barnes peptide MALLLAVF in front of the full-length P450 sequence (Barnes), (4) substitution of the first 8 amino acids with Barnes peptide MALLLAVF, (5) addition of 28 amino acid synthetic peptide in front of the full-length P450 sequence (28aa), (6) substitution of the native transmembrane domain with the transmembrane domain of SohB probable protease from *E. coli* (SohB), and (7) addition of *E. coli* outer membrane protein A (OmpA) signal peptide in front of the full-length P450 sequence (OmpA). 28aa, SohB and OmpA sequences were ordered from Twist Bioscience as short DNA fragments, amplified with uracil-containing primers, and used to create modified P450s on a separate plasmid backbone before they were cloned into the expression vector. Barnes sequence was short enough to be inserted into the primer overhangs that were used to create the modifications (3) and (4), and truncated P450s were prepared with primers annealing at within the gene sequences. DNA sequences of all used modifications are shown in Table S4.

### Fermentation conditions

Overnight cultures were started by picking single colonies into 1.2 mL LB media with appropriate antibiotics in a 24-well plate. Then, the overnight cultures were diluted 1000-fold into 1.2 mL autoinducing medium in round bottom 24-well plates and the fermentation was carried out in a rotary shaker at 18 °C, 200 RPM for 72 h. Autoinducing medium, adapted from Studier et al. [42], was used during all fermentations unless stated otherwise. The original recipe was adjusted to match Terrific broth (TB) in the content of yeast extract and tryptone and in the buffer composition. The TB-based autoinducing medium contained 24 g/L yeast extract, 12 g/L tryptone, 100 mM KH_2_PO_4_-K_2_HPO_4_ buffer pH 7.0, 0.05% glucose, 0.5% glycerol, 0.2% α-lactose, 2 mM MgSO_4_, and trace metal mix (50 µM FeCl_3_, 20 µM CaCl_2_, 10 µM MnCl_2_, 10 µM ZnSO_4_, 2 µM CoCl_2_, 2 µM CuCl_2_, 2 µM NiCl_2_, 2 µM Na_2_MoO_4_, 2 µM Na_2_SeO_3_, 2 µM H_3_BO_3_). To improve heme biosynthesis necessary for functional P450 enzymes, 0.5 mM 5-aminolevulinic acid was supplied to the media. Corresponding antibiotics were also added at the following concentrations: carbenicillin (50 µg/mL), spectinomycin (50 µg/mL), kanamycin (50 µg/mL) and chloramphenicol (34 µg/mL). 1 mM L-homophenylalanine (TCI Chemicals, Tokyo, Japan) was supplemented to the strain expressing *Bv*CYP79F6. Glutathione is one of the most abundant metabolites in *E. coli* [43] and as such was not supplied in the media above the levels found in yeast extract.

M9 minimal media recipe was adjusted to allow for autoinduction of protein expression, and supplemented with 5-aminolevulinic acid, thiamine and trace metals. The final composition was 12.8 g/L Na_2_HPO_4_.7H_2_O, 3 g/L KH_2_PO_4_, 1 g/L NH_4_Cl, 0.5 g/L NaCl, 2 mM MgSO_4_, 1mM thiamine, 0.1 mM CaCl_2_, 0.5 mM 5-aminolevulinic acid, 0.05% glucose, 0.5% glycerol, 0.2% α-lactose and trace metal mix (50 µM FeCl_3_, 20 µM CaCl_2_, 10 µM MnCl_2_, 10 µM ZnSO_4_, 2 µM CoCl_2_, 2 µM CuCl_2_, 2 µM NiCl_2_, 2 µM Na_2_MoO_4_, 2 µM Na_2_SeO_3_, 2 µM H_3_BO_3_).

### Metabolomics and targeted proteomics analyses

Media and cell samples were collected separately after the cells were spun down from 1 mL cultures in deep 96-well plates at 3700 x *g* for 5 min. The media samples were stored at − 20 °C and the cell pellet at − 80 °C until analysis. For metabolite analysis an aliquot of the media was diluted 25-fold in water and filtered through 0.22 μm filters. The glutathione conjugates derived from Tyr, Trp, Leu, Val and HPhe were analysed with Q-TOF LC-MS/MS using their characteristic fragmentation pattern as described previously [36, 37] (Fig. S6). Phe-Ox and Phe-GSH were analysed by triple quadrupole LC-MS/MS after diluting the media further 10-fold with ^13^ C-,^15^ N-labeled amino acid mix (250-fold total dilution, 10 µg/ml, Isotec, Miamisburg, US). The titres were quantified with ^13^ C-, ^15^ N-Phe as internal standard using response factors calculated from dilution series of the analytes in spent *E. coli* media to account for the matrix effect (Table S6). Outliers were defined as data lower than Q1 – 2 * IQR and higher than Q3 + 2 * IQR, and were removed from the dataset, where IQR is interquartile range and Q1 and Q3 are lower and upper quartiles, respectively. Student’s upper-tailed *t* test was performed to identify engineered variants with significant increase in product generation compared to the native enzymes. Statistical analysis and visualisation were done using R (version 4.2.2, [44]) in RStudio 2022.07.2.576 [45] relying on tidyverse [46] and ggplot2 [47] packages. For targeted proteomics, the cells were disrupted with BugBuster Protein Extraction Reagent (Merck, Darmstadt, Germany) and the sample preparation method was adapted from Batth et al. [48] as previously described [28].

### Electronic supplementary material

Below is the link to the electronic supplementary material.

Supplementary Material 1

## Data Availability

The data supporting the conclusions of the study are included in the supplementary material.
