# Supplementary material for: Systematic engineering pinpoints a versatile strategy for the expression of functional cytochrome P450 enzymes in Escherichia coli cell factories
Source: Microb Cell Fact. 2023 Oct 25;22:219. doi: 10.1186/s12934-023-02219-7 (PMC10601251; doi:10.1186/s12934-023-02219-7)
Supplement: Supplementary file 1 — Supplementary Material 1 [file 12934_2023_2219_MOESM1_ESM.docx]

**Supplementary Material for**

Systematic engineering pinpoints a universal strategy for the expression of functional cytochrome P450 enzymes in Escherichia coli

Michal Poborsky, Christoph Crocoll, Mohammed Saddik Motawia and Barbara Ann Halkier

**Corresponding author:**

Barbara Ann Halkier

bah@plen.ku.dk

**This PDF file includes:**

Supplementary materials and methods

Figures S1 to S6

Tables S1 to S8

SI References

Supplementary Materials and Methods

**Oligonucleotides, genes, plasmids, and bacterial strains**

Oligonucleotides for cloning and sequence validation were purchased from Integrated DNA Technologies (IDT, Leuven, Belgium), Sanger sequencing was provided by Eurofins Genomics (Ebersberg, Germany) and DNA preparation was performed with bacterial kits from Omega Bio-Tek (Norcross, GA, USA). Plant genes were ordered codon-optimized from IDT and Twist Bioscience (San Francisco, CA, USA). All plasmids used in the study are listed in Table S7 and the complete annotated plasmid sequences were uploaded to an online repository (<https://doi.org/10.6084/m9.figshare.24164154>). Proofreading Phusion U Hot Start DNA polymerase was used to amplify all DNA parts and DreamTaq DNA polymerase for colony PCR (both Thermo Fisher Scientific, Waltham, USA). Restriction enzymes and USER enzyme mix were purchased from New England BioLabs. pET-52b (Novagen®, Merck, #71554) and pCDF-1b (Novagen®, Merck, #71330) plasmids were used to express the heterologous genes. *E. coli* strains were purchased from New England BioLabs (Ipswich, MA, USA). DNA cloning was performed with *E. coli* NEB 10-β strain and fermentations were done with *E. coli* BL21 (DE3) strain. In all experiments, the producing strain was transformed with pRI952 plasmid carrying genes for rare tRNAs for isoleucine and arginine [1].

**Transformation of the expression strains**

Allowing the transformation of 3 plasmids at the same time, electrocompetent cells were always prepared fresh instead of using frozen aliquots. 30 µL of overnight culture were inoculated into 1.4 mL LB medium in a microcentrifuge tube and grown for 3-4 hours at 37 °C, 900 RPM in a shaking heating block. Afterwards, the cells were washed twice with 1 mL of ice-cold water and transformed with 1 µL of each plasmid (~ 100 ng). GenePulser Xcell electroporator (Bio-Rad, Hercules, CA, USA) was set to 1350 V, 10 µF, 600 Ohms. The transformants were recovered in LB medium supplemented with 0.4 % glucose for 1 h at 37 °C, 900 RPM, spread on LB agar with appropriate antibiotics, and grown overnight at 37 °C.

**Combined analysis of amino acids, phenylacetaldoxime and phenylacetaldoxime glutathione conjugate by LC coupled to triple quadrupole MS (LC-MS/QqQ).**

The pre-diluted media samples were diluted further in a ratio of 1:10 (v:v) in water containing the ^13^C-, ^15^N-labeled amino acid mix (10 µg/mL, Isotec, Miamisburg, US). Amino acids in the diluted extracts were directly analysed by LC-MS/MS. The analysis method originates from a protocol described by Jander et al. [2] and was modified from the protocol described in Docimo et al. [3] to adjust for instrument settings and inclusion of metabolites from the benzyl-glucosinolate pathway. Chromatography was performed on an Advance ultra-high-performance liquid chromatography (UHPLC) system (Bruker, Bremen, Germany). Separation was achieved on a Zorbax Eclipse XDB-C18 column (50 x 4.6mm, 1.8µm, Agilent Technologies, Germany). Formic acid (0.05%) in water (v/v) and acetonitrile (supplied with 0.05% formic acid, v/v) were employed as mobile phases A and B, respectively. The elution profile was: 0-1.2 min, 3 % B; 1.2-3.8 min, 3-65 % B; 3.8-4.0 min 65-100% B, 4.0-4.6 min 100% B, 4.6-4.7 min 100-3% B and 4.7-6.0 min in 3% B. The mobile phase flow rate was 500 µl/min. The column temperature was maintained at 40 °C. The liquid chromatography was coupled to an EVOQ Elite Triplequadrupole (QqQ) mass spectrometer (Bruker, Bremen, Germany) equipped with an electrospray ion source (ESI) operated in combined positive and negative ionization modes. The instrument parameters were optimized by infusion experiments with pure standards. The ion spray voltage was maintained at +3000 V or -4000V for amino acid and glucosinolate analysis, respectively. The cone temperature was set to 300 °C and the cone gas to 20 psi. The heated probe temperature was set to 300 °C and the probe gas flow to 50 psi. Nebulizing gas was set to 60 psi and collision gas to 1.6 mTorr. Nitrogen was used as the probe and nebulizing gas and argon as the collision gas. The active exhaust was constantly on. Multiple reaction monitoring (MRM) was used to monitor analyte parent ion → product ion transitions: MRMs were chosen [2–5]

Bruker MS Workstation software (Version 8.2.1, Bruker, Bremen, Germany) was used for data acquisition and processing. Linearity in ionization efficiencies was verified by analysing dilution series of standard mixtures (amino acid standard mix, Fluka plus Gln and Trp, also Fluka). All samples were spiked with ^13^C-, ^15^N-labeled amino acids at a concentration of 10 µg/mL. The concentration of the individual labelled amino acids in the mix had been determined by comparison to a reference standard by LC-MS/MS analysis. Individual amino acids in the sample were quantified by the respective ^13^C-, ^15^N-labeled amino acid internal standards, for tryptophan and asparagine and glutamine: tryptophan was quantified using ^13^C-, ^15^N-Phe applying a response factor of 0.42, asparagine and glutamine were quantified using ^13^C-, ^15^N-Asp and ^13^C-, ^15^N-Glu applying a response factor of 1.0. Additional MRMs for glucosinolate intermediates like Phe-Ox and Phe-GSH were included into the method.

**Analysis of phenylacetaldoxime in M9 minimal media by LC-MS/QqQ.**

Media samples were diluted 10-fold with deionized water and further 10-fold in water containing the 13C-, 15N-labeled amino acid mix (10 µg/mL), filtered (Millipore 0.22 µm filter plate) and subjected to analysis by LC-MS/MS. Briefly, chromatography was performed on a 1290 Infinity II UHPLC system (Agilent Technologies). Separation was achieved on a Zorbax RRHD Eclipse XDB-C18 column (150 x 3.0 mm, 1.8 µm, Agilent Technologies). Formic acid (0.05%, v/v) in water and acetonitrile (supplied with 0.05% formic acid, v/v) were employed as mobile phases A and B respectively. The elution profile for was: 0.0-0.75 min, 3 % B; 0.75-5.1 min, 3-90 % B; 5.1-5.2 min 90-100 % B, 5.2-5.95 min 100 % B, 5.95-6.0 min, 100-3 % B and 6.0-7.0 min 3 % B. The mobile phase flow rate was 400 µL/min. The column temperature was maintained at 30 °C. The liquid chromatography was coupled to an Ultivo Triplequadrupole mass spectrometer (Agilent Technologies) equipped with a Jetstream electrospray ion source (ESI). The ion spray voltage was set to +2500 V in positive ion mode. Dry gas temperature was set to 325 °C and dry gas flow to 11 L/min. Sheath gas temperature was set to 350 °C and sheath gas flow to 12 L/min. Nebulizing gas was set to 40 psi. Nitrogen was used as dry gas, nebulizing gas and collision gas. The instrument parameters were optimized for best detection of all metabolites with mixes of reference standards. Multiple reaction monitoring (MRM) was used to monitor precursor ion → fragment ion transitions. MRM transitions were identical to the transitions used in the method described Table S6. Both Q1 and Q3 quadrupoles were maintained at unit resolution. Mass Hunter Quantitation Analysis for QQQ software (Version 10.1, Agilent Technologies) was used for data processing. Triplicate injections of dilution series were used for quantification.

**Analysis of glutathione conjugates by Q-TOF LC-MS/MS.**

Samples for Q-TOF analysis were prepared similarly to the triple quad, but at higher concentrations, only diluting the media 25-fold in water spiked with 1 µM caffeine as internal standard. To identify potential intermediates not included in the targeted identification by LC-MS/QqQ as described above, samples were also subjected to untargeted analysis by LC-MS/Q-TOF. Chromatography was performed on a Dionex UltiMate® 3000 Quaternary Rapid Separation UHPLC^+^ focused system (Thermo Fisher Scientific, Germering, Germany). Separation was achieved on a Kinetex 1.7u XB-C18 column (100 x 2.1 mm, 1.7 µm, 100 Å, Phenomenex, Torrance, CA, USA). Formic acid (0.05%) in water and acetonitrile (supplied with 0.05% formic acid) were employed as mobile phases A and B, respectively. The elution profile was: 0.00-0.1 min, 2% B; 0.1.-16.0 min, 2-45% B; 16.0-24.5 min 45-100% B, 24.5-26.5 min 100% B, 26.5-26.55 min 100-2% B and 26.55-30.0 2% B. The mobile phase flow rate was 300 µl/min. The column temperature was maintained at 25 °C. The liquid chromatography was coupled to a Compact micrOTOF-Q mass spectrometer (Bruker, Bremen, Germany) equipped with an ESI operated in positive mode. Settings for positive ion mode were as follows: the ion spray voltage was maintained at +4500 V. Dry temperature was set to 250 °C and dry gas flow was set to 8 L/min. Nebulizing gas was set to 2.5 bar and collision energy to 10 eV. Nitrogen was used as dry gas, nebulizing gas, and collision gas. Sodium formate (Na-formate) clusters were used as calibrant AutoMSMS mode employed to collect MS and MS/MS spectra of the three most abundant ions present. The acquisition rate was at 2 Hz. The *m/z* range was set to 50-1000 for MS acquisition and *m/z* 200-800 for MS/MS acquisition. All files were automatically calibrated based on the compound spectra collected from the Na-formate clusters by post-processing.

**Targeted proteomic analysis by triple quadrupole LC-MS/MS.**

The sample preparation and LC-MS/MS conditions were adopted with changes from previous reports [4,6,7] and optimized for the LC-MS/MS system used in this study. Briefly, MilliQ grade water with 0.05 % formic acid and acetonitrile with 0.05 % formic acid were used as solvent A and B, respectively. Flow rate was 400 uL/min with the following gradient conditions: 0.0-0.5 min 5 % B; 0.5-22.0 min 5-32 % B; 22.0-24.0 min 32-42 % B; 24-25 min 42-90 % B; 25.0-28.0 min 90% B; 28.0-31.0 min 90-5 % B; 31.0-35.0 min 5 % B. Column oven temperature was maintained at 55°C Peptide separation was achieved on an Aeris™ 2.6 µm PEPTIDE XB-C18 100 Å, 150 x 2.1 mm (Phenomenex). The injection volume was 15 µL. The column temperature was maintained at 55 °C. The liquid chromatography was coupled to an Ultivo Triplequadrupole mass spectrometer (Agilent Technologies) equipped with a Jetstream electrospray ion source (ESI) operated in positive ion mode. Instrument parameters were optimized by running different mixes of the target peptides. Mixes of the target peptides were also used to identify the three most abundant transitions from analyte precursor ion → fragment ion transitions for selected reaction monitoring (SRM). Collision energy values were identical for light and heavy peptide transitions pairs. Fragmentor voltage was set to 130 V for all peptides. Source settings for heated electrospray ionization were as follows: spray voltage 3000 V, positive ion mode; gas temperature 325 °C; gas flow 13 L/min; nebulizer 25 psi; sheath gas temperature 400 °C; and sheath gas flow 12 L/min. The triple quadrupole mass spectrometer (Ultivo, Agilent Technologies) was set to scan for transitions for individual peptides within scheduled 2 min windows (Table S7). Quadrupoles 1 and 3 were set to unit resolution. Non-injections were removed from the data. The acquired chromatograms were analysed through Skyline 22. 2.0.312.


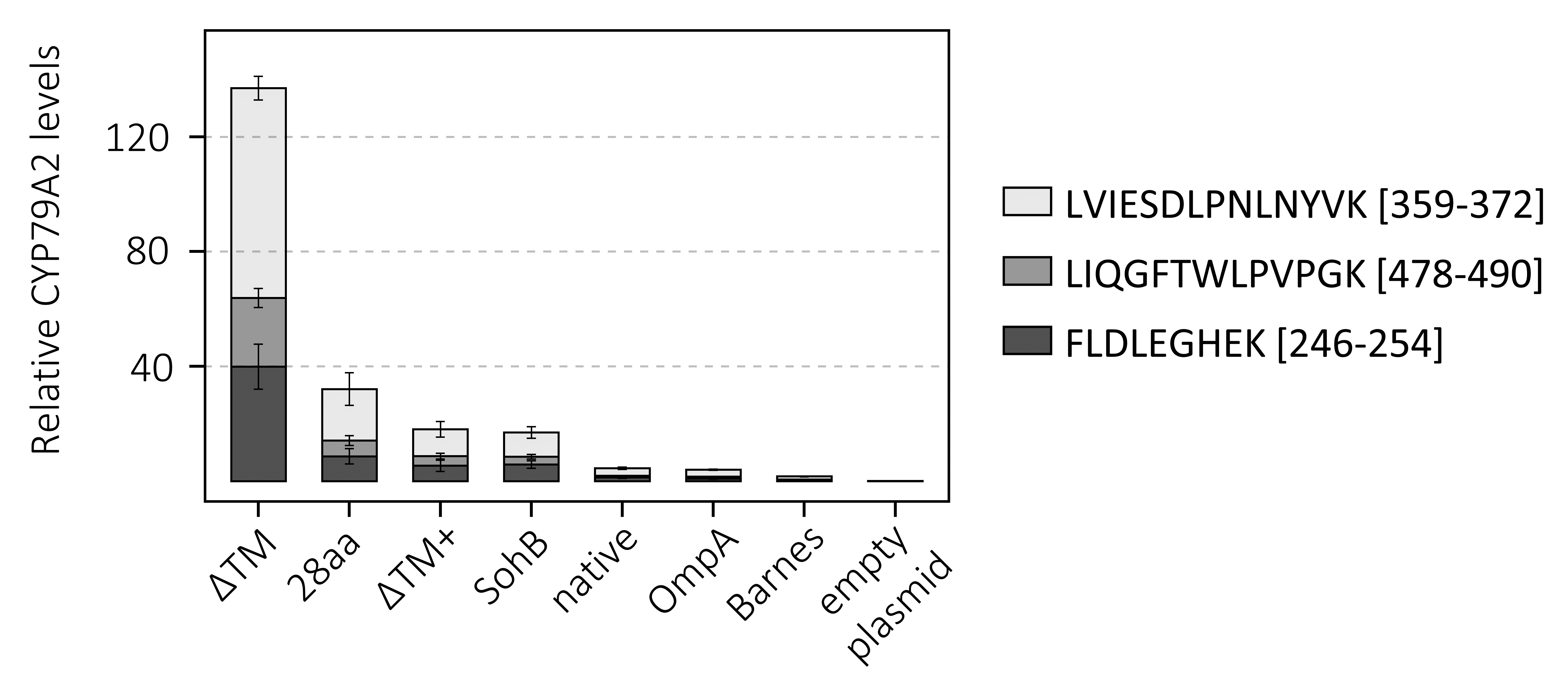
Supplementary Figures

Fig. S1. Expression levels of CYP79A2 represented by three proteotypic peptides normalised to the expression of *E. coli* isocitrate dehydrogenase. The bars represent the mean of 3-6 biological (non-injections during LC/MS) and error bars represent standard error of the mean.


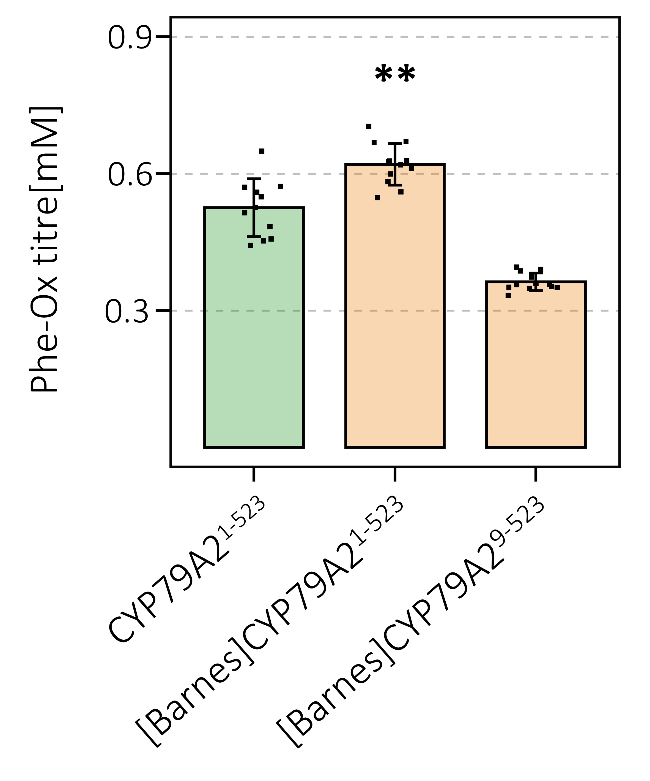


Fig. S2. Insertion of MALLLAVF peptide in front of full-length CYP79A2 or by substituting the first eight amino acids. Every strain was grown in 12 biological replicates and the error bars represent standard deviation from the mean. Student’s two-tailed *t* test notes significant increase in Phe-Ox titre compared to native with p value (with Holm adjustment), ** p < 0.01.


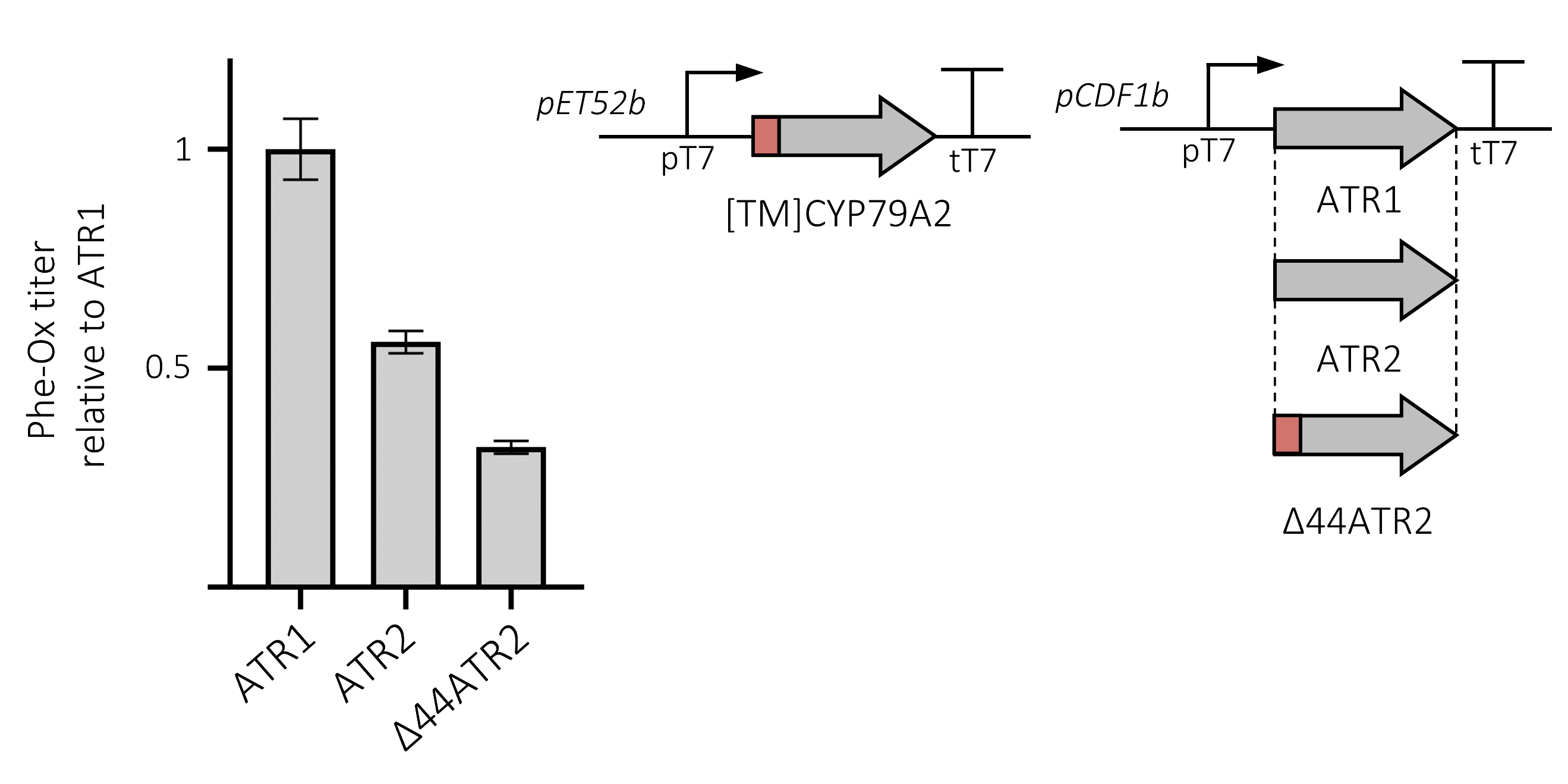


Fig. S3. Testing of the two available cytochrome P450 reductases from *A. thaliana*, ATR1, ATR2 and its truncated variant Δ44ATR2. The protein expression was induced by addition of 0.5 mM Isopropyl ß-D-1-thiogalactopyranoside (IPTG) after the cultures reached OD600 of 0.6. Every strain was grown in 3 biological replicates and the error bars represent standard deviation from the mean.


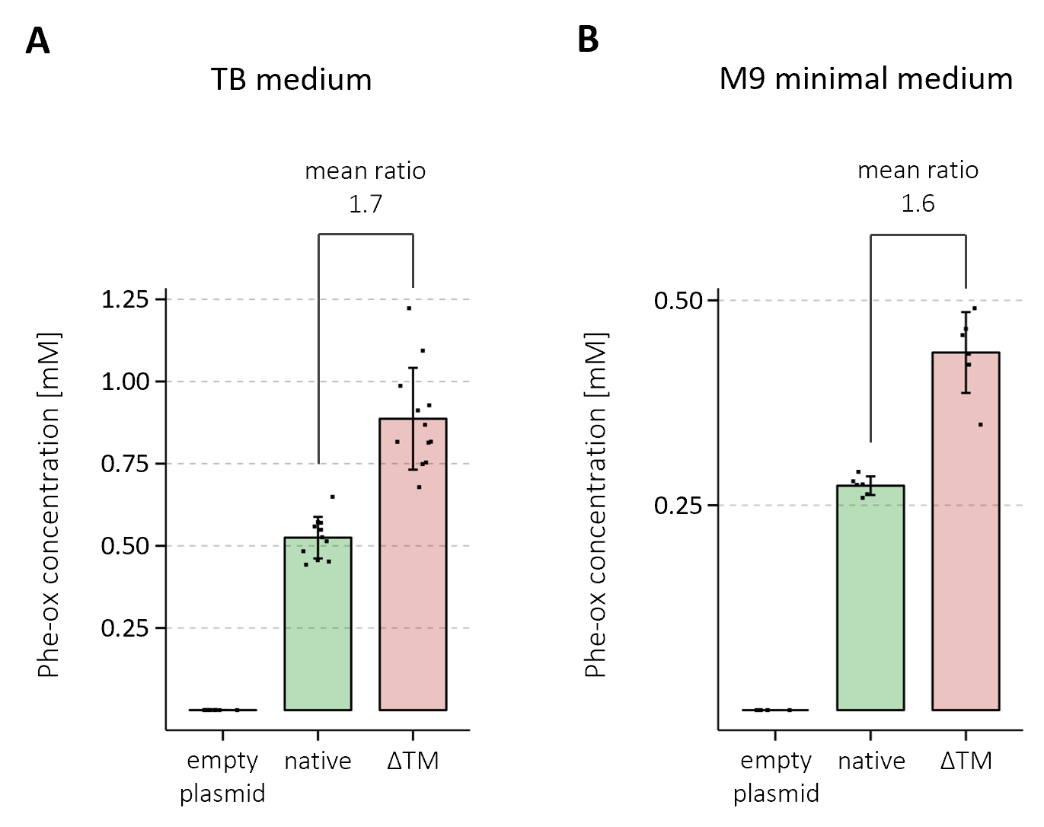


Fig. S4. The comparison of the N-terminal truncation effect between rich TB media (A) and supplemented M9 minimal media (B). Every strain was grown in 12 (TB media) or 6 (M9 minimal media) biological replicates and the error bars represent standard deviation from the mean.


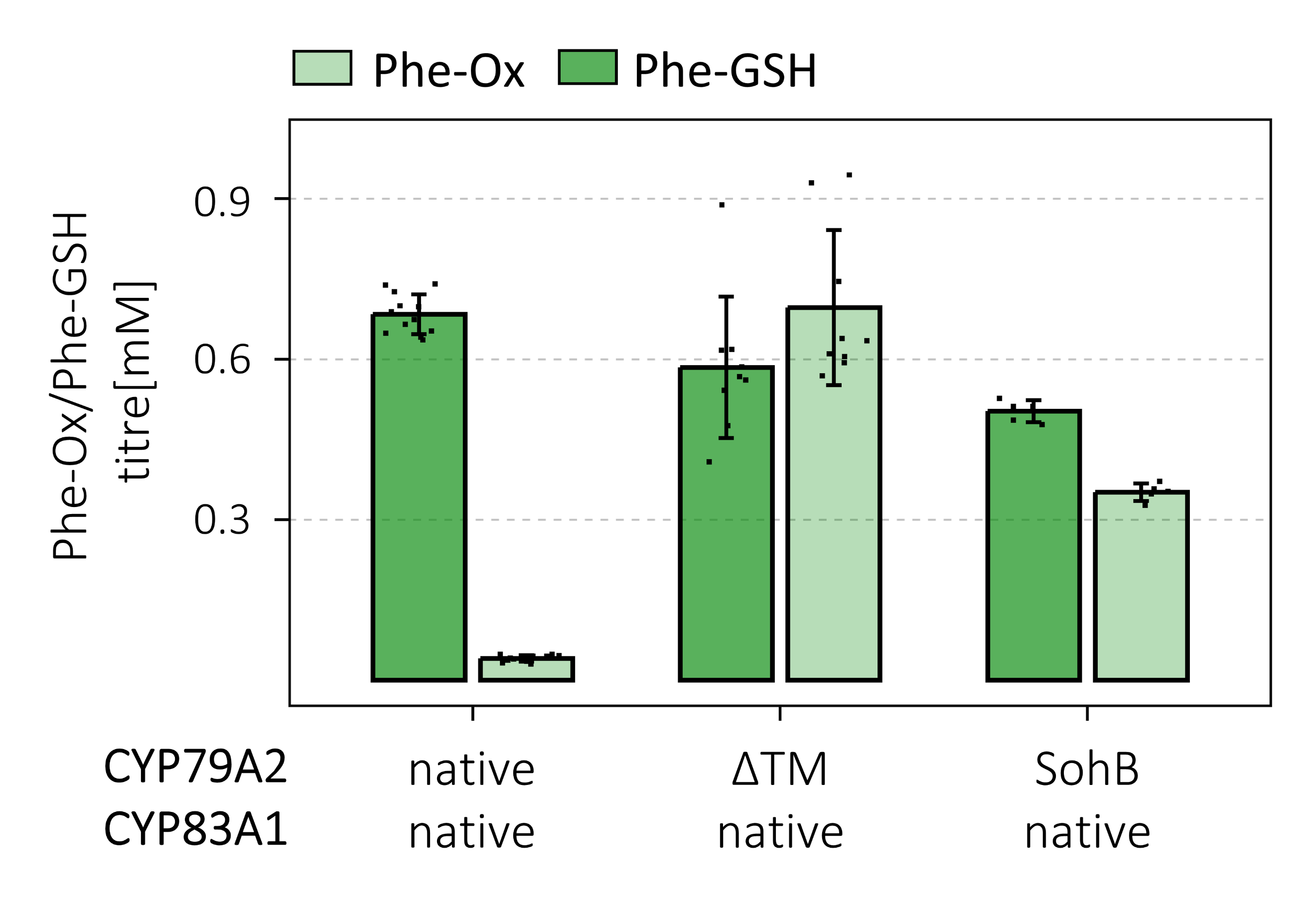


Fig. S5. Comparison of pairing native CYP83A1 with 2 different engineered variants of CYP79A2 in a similar setup as Fig 3D in the main text. Every strain was grown in 6 (SohB) or 12 (native and ΔTM) biological replicates and the error bars represent standard deviation from the mean.


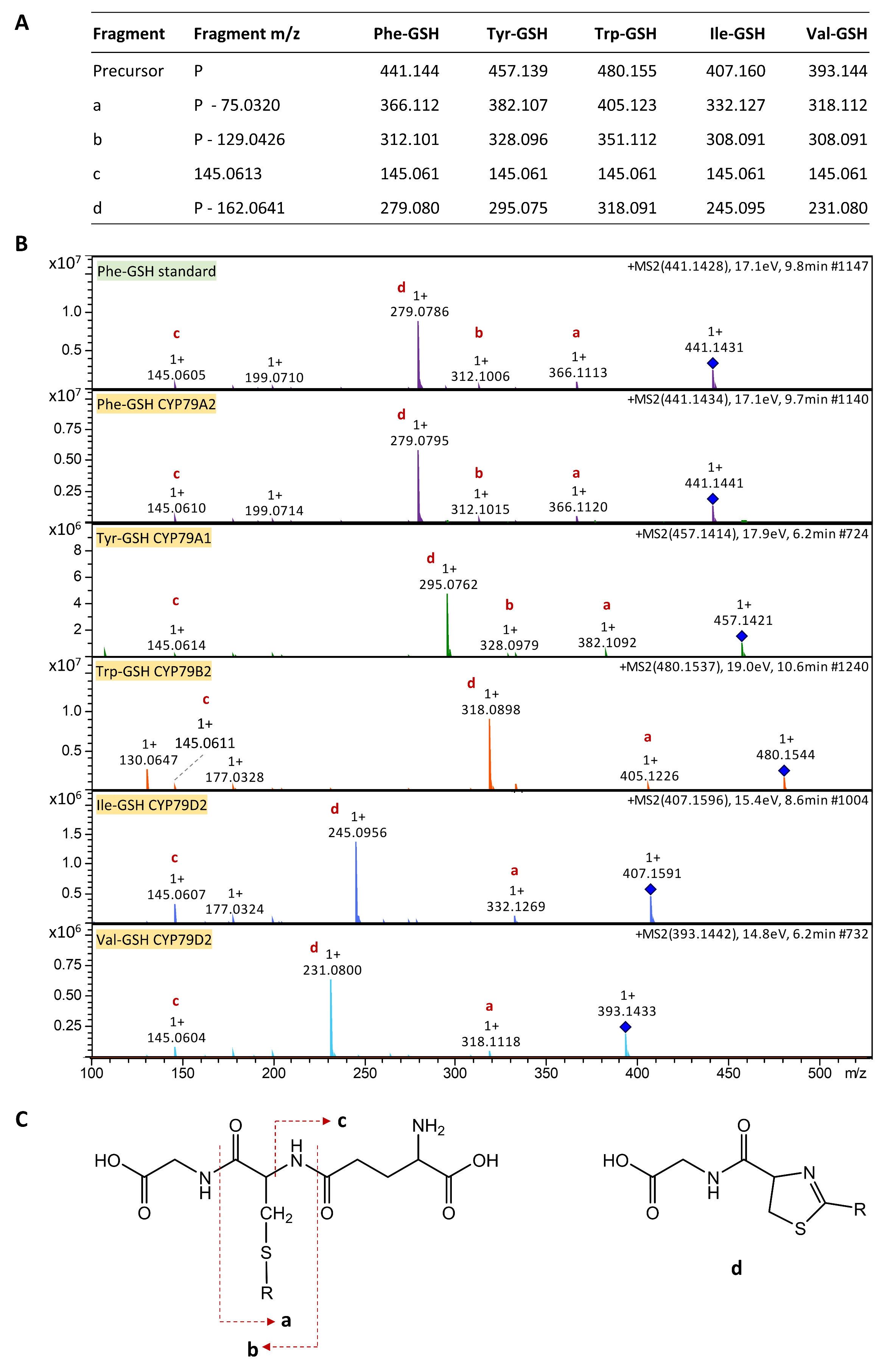


Fig. S6. A Glutathione-conjugate characteristic fragments [8,9] with masses corresponding to intermediates derived from specific amino acids. B The fragments are demonstrated using an authentic standard of GSH-conjugate with phenylacetonitrile oxide, an intermediate of benzyl glucosinolate biosynthesis, and then our samples upon expression of different CYP79s. C Molecular fragments of glutathione conjugates detected in our experiments.


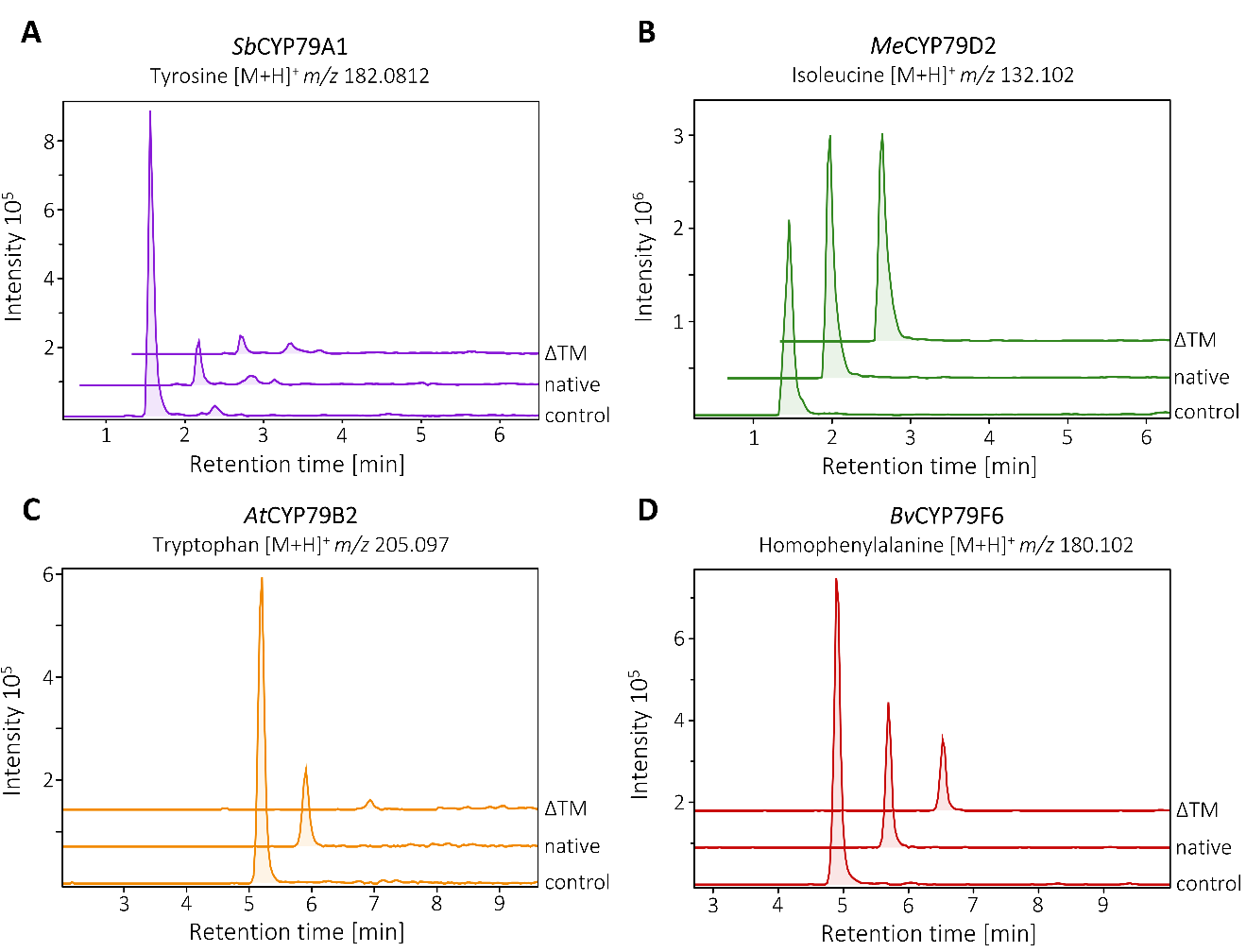


Fig. S7. Extracted ion chromatograms comparing the substrate consumption between native and N-terminally truncated *Sb*CYP79A1 (A), *Me*CYP79D2 (B), *At*CYP79B2 (C), *Bv*CYP79F6 (D) and a control without expression P450s.

Supplementary Tables

Table S1. The N-terminal sequence modifications affect in vivo production of phenylacetaldoxime (Phe-Ox). The table provides additional information to results in Fig. 2., showing the exact concentrations of the product and substrate as well as the optical density (600 nm) of the culture after 72 hours of fermentation.

| **Strain** | **Phe-Ox [mM]** | **Phe [mM]** | **OD600 [a.u.]** |
| --- | --- | --- | --- |
| empty plasmids | 0.00 ± 0.00 | 4.69 ± 0.47 | 15.2 ± 0.9 |
| native CYP79A2 | 0.52 ± 0.06 | 4.07 ± 0.33 | 15.2 ± 0.6 |
| [ΔTM]CYP79A2 | 0.89 ± 0.15 | 3.49 ± 0.38 | 15.9 ± 2.1 |
| [ΔTM+]CYP79A2 | 0.63 ± 0.05 | 4.18 ± 0.20 | 14.8 ± 1.6 |
| [28aa]CYP79A2 | 0.32 ± 0.06 | 4.27 ± 0.66 | 17.1 ± 0.7 |
| [Barnes]CYP79A2 | 0.62 ± 0.05 | 3.88 ± 0.25 | 16.8 ± 1.7 |
| [SohB]d14CYP79A2 | 0.69 ± 0.09 | 3.78 ± 0.36 | 17.3 ± 1.2 |
| [OmpA]CYP79A2 | 0.22 ± 0.06 | 5.33 ± 0.52 | 11.5 ± 3.8 |

Errors correspond to the standard deviation from the mean.
All data represented by 12 biological replicates.

Table S2. The changes in CYP79A2 protein expression levels upon N-terminal engineering. The CYP79A2 levels were normalized to the levels of isocitrate dehydrogenase as in Fig. 2C.

| **Strain** | **Relative CYP79A2 levels [a.u.]** | **n** |
| --- | --- | --- |
| empty plasmids | 0.0 ± 0.1 | 5 |
| native CYP79A2 | 2.6 ± 0.9 | 4 |
| [ΔTM]CYP79A2 | 73.1 ± 7.1 | 3 |
| [ΔTM+]CYP79A2 | 9.3 ± 5.5 | 4 |
| [28aa]CYP79A2 | 17.9 ± 12.7 | 5 |
| [Barnes]CYP79A2 | 1.1 ± 0.3 | 4 |
| [SohB]d14CYP79A2 | 8.4 ± 3.5 | 3 |
| [OmpA]CYP79A2 | 2.4 ± 0.4 | 5 |

Errors correspond to the standard deviation from the mean.

Table S3. Concurrent N-terminal sequence modifications of CYP79A2 and CYP83B1/CYP83A1 affects in vivo production of phenylacetaldoxime (Phe-Ox) and phenylacetaldoxime glutathione conjugate (Phe-GSH). The table provides additional information to results in Fig. 3., showing the exact concentrations of the product and substrate as well as the optical density (600 nm) of the culture after 72 hours of fermentation.

| **CYP79A2** | **CYP83B1** | **Phe-Ox titre [mM]** | | **Phe-GSH titre [mM]** | **Phe [mM]** | **OD600 [a.u.]** |
| --- | --- | --- | --- | --- | --- | --- |
| native | native | 0.02 ± 0.00 | | 0.76 ± 0.10 | 3.82 ± 0.34 | 18.6 ± 1.0 |
| ΔTM | native | 0.38 ± 0.06 | | 0.57 ± 0.06 | 3.68 ± 0.47 | 19.5 ± 2.1 |
| ΔTM | ΔTM | 0.04 ± 0.01 | | 1.42 ± 0.04 | 3.77 ± 0.07 | 20.6 ± 1.4 |
| ΔTM | ΔTM+ | 0.63 ± 0.13 | | 0.24 ± 0.03 | 3.76 ± 0.37 | 20.2 ± 2.6 |
| ΔTM | 28aa | 0.58 ± 0.11 | | 0.48 ± 0.07 | 3.46 ± 0.40 | 18.1 ± 2.0 |
| ΔTM | Barnes | 0.08 ± 0.02 | | 1.30 ± 0.11 | 3.78 ± 0.45 | 18.6 ± 2.2 |
| ΔTM | SohB | 0.04 ± 0.01 | | 1.47 ± 0.30 | 3.82 ± 0.48 | 20.6 ± 1.3 |
| ΔTM | OmpA | 0.11 ± 0.04 | | 1.43 ± 0.08 | 3.86 ± 0.63 | 18.7 ± 1.9 |
| **CYP79A2** | **CYP83A1** | | **Phe-Ox titre [mM]** | **Phe-GSH titre [mM]** | **Phe [mM]** | **OD600 [a.u.]** |
| native | native | | 0.04 ± 0.01 | 0.68 ± 0.04 | 3.96 ± 0.54 | 18.9 ± 1.4 |
| ΔTM | native | | 0.70 ± 0.14 | 0.58 ± 0.13 | 4.41 ± 1.21 | 17.2 ± 2.6 |
| ΔTM | ΔTM | | 0.05 ± 0.01 | 1.47 ± 0.28 | 4.05 ± 0.31 | 21.0 ± 1.6 |
| ΔTM | ΔTM+ | | 0.86 ± 0.09 | 0.21 ± 0.03 | 3.08 ± 0.43 | 18.2 ± 1.5 |
| ΔTM | 28aa | | 0.87 ± 0.12 | 0.06 ± 0.01 | 2.69 ± 0.17 | 21.3 ± 3.3 |
| ΔTM | Barnes | | 0.33 ± 0.06 | 0.73 ± 0.11 | 3.92 ± 0.37 | 15.6 ± 2.5 |
| ΔTM | SohB | | 0.37 ± 0.05 | 0.66 ± 0.11 | 3.66 ± 0.43 | 18.9 ± 1.5 |
| ΔTM | OmpA | | 0.36 ± 0.02 | 0.67 ± 0.10 | 4.58 ± 0.41 | 13.9 ± 2.6 |

Errors correspond to the standard deviation from the mean.
All data represented by 12 biological replicates.

Table S4. Nucleotide sequences of all P450 modifications used in the study other than truncation.

| **Modification** | **Sequence 5'-> 3'** |
| --- | --- |
| Barnes | ATGGCTCTGTTATTAGCAGTTTTT |
| 28aa | ATGGAATTATCACAAGTTTGTACAAAAAAGGAGGCTGGCGCCGGAACCAATTCAGTCGACTGGATCCAAGAAGGAGATATAACC |
| SohB | ATGGAATTGTTGTCTGAATATGGTTTGTTTTTGGCGAAAATCGTTACCGTTGTGCTAGCGATTGCGGCGATTGCCGCCATTATTGTCAATGTTGCTCAACGTAATAAACGCCAGCGTGGCGAGTTACGGGTAAACAATCTTAGC |
| OmpA | ATGAAAAAGACAGCTATCGCGATTGCAGTGGCACTGGCTGGTTTCGCTACCGTAGCTCAGGCC |

Table S5. The amino acid sequences of all seven engineered cytochrome P450 enzymes. The truncations are highlighted in the sequence with ΔTM represented in red with underline and ΔTM+ in red.

| *At*CYP79A2 523\|509\|500 amino acids [native\|ΔTM\|ΔTM+] |
| --- |
| MLAFIIGLLLLALTMKRKEKKKTMLISPTRNLSLPPGPKSWPLIGNLPEILGRNKPVFRWIHSLMKELNTDIACIRLANTHVIPVTSPRIAREILKKQDSVFATRPLTMGTEYCSRGYLTVAVEPQGEQWKKMRRVVASHVTSKKSFQMMLQKRTEEADNLVRYINNRSVKNRGNAFVVIDLRLAVRQYSGNVARKMMFGIRHFGKGSEDGSGPGLEEIEHVESLFTVLTHLYAFALSDYVPWLRFLDLEGHEKVVSNAMRNVSKYNDPFVDERLMQWRNGKMKEPQDFLDMFIIAKDTDGKPTLSDEEIKAQVTELMLATVDNPSNAAEWGMAEMINEPSIMQKAVEEIDRVVGKDRLVIESDLPNLNYVKACVKEAFRLHPVAPFNLPHMSTTDTVVDGYFIPKGSHVLISRMGIGRNPSVWDKPHKFDPERHLSTNTCVDLNESDLNIISFSAGRRGCMGVDIGSAMTYMLLARLIQGFTWLPVPGKNKIDISESKNDLFMAKPLYAVATPRLAPHVYPT* |
| *At*CYP83A1 502\|482\|474 amino acids [native\|ΔTM\|ΔTM+] |
| MEDIIIGVVALAAVLLFFLYQKPKTKRYKLPPGPSPLPVIGNLLQLQKLNPQRFFAGWAKKYGPILSYRIGSRTMVVISSAELAKELLKTQDVNFADRPPHRGHEFISYGRRDMALNHYTPYYREIRKMGMNHLFSPTRVATFKHVREEEARRMMDKINKAADKSEVVDISELMLTFTNSVVCRQAFGKKYNEDGEEMKRFIKILYGTQSVLGKIFFSDFFPYCGFLDDLSGLTAYMKECFERQDTYIQEVVNETLDPKRVKPETESMIDLLMGIYKEQPFASEFTVDNVKAVILDIVVAGTDTAAAAVVWGMTYLMKYPQVLKKAQAEVREYMKEKGSTFVTEDDVKNLPYFRALVKETLRIEPVIPLLIPRACIQDTKIAGYDIPAGTTVNVNAWAVSRDEKEWGPNPDEFRPERFLEKEVDFKGTDYEFIPFGSGRRMCPGMRLGAAMLEVPYANLLLSFNFKLPNGMKPDDINMDVMTGLAMHKSQHLKLVPEKVNKY* |
| *At*CYP83B1 499\| 477\|472 amino acids [native\|ΔTM\|ΔTM+] |
| MDLLLIIAGLVAAAAFFFLRSTTKKSLRLPPGPKGLPIIGNLHQMEKFNPQHFLFRLSKLYGPIFTMKIGGRRLAVISSAELAKELLKTQDLNFTARPLLKGQQTMSYQGRELGFGQYTAYYREMRKMCMVNLFSPNRVASFRPVREEECQRMMDKIYKAADQSGTVDLSELLLSFTNCVVCRQAFGKRYNEYGTEMKRFIDILYETQALLGTLFFSDLFPYFGFLDNLTGLSARLKKAFKELDTYLQELLDETLDPNRPKQETESFIDLLMQIYKDQPFSIKFTHENVKAMILDIVVPGTDTAAAVVVWAMTYLIKYPEAMKKAQDEVRSVIGDKGYVSEEDIPNLPYLKAVIKESLRLEPVIPILLHRETIADAKIGGYDIPAKTIIQVNAWAVSRDTAAWGDNPNEFIPERFMNEHKGVDFKGQDFELLPFGSGRRMCPAMHLGIAMVEIPFANLLYKFDWSLPKGIKPEDIKMDVMTGLAMHKKEHLVLAPTKHI* |
| *At*CYP79B2 541\|500 amino acids [native\|ΔTM] |
| MNTFTSNSSDLTTTATETSSFSTLYLLSTLQAFVAITLVMLLKKLMTDPNKKKPYLPPGPTGWPIIGMIPTMLKSRPVFRWLHSIMKQLNTEIACVKLGNTHVITVTCPKIAREILKQQDALFASRPLTYAQKILSNGYKTCVITPFGDQFKKMRKVVMTELVCPARHRWLHQKRSEENDHLTAWVYNMVKNSGSVDFRFMTRHYCGNAIKKLMFGTRTFSKNTAPDGGPTVEDVEHMEAMFEALGFTFAFCISDYLPMLTGLDLNGHEKIMRESSAIMDKYHDPIIDERIKMWREGKRTQIEDFLDIFISIKDEQGNPLLTADEIKPTIKELVMAAPDNPSNAVEWAMAEMVNKPEILRKAMEEIDRVVGKERLVQESDIPKLNYVKAILREAFRLHPVAAFNLPHVALSDTTVAGYHIPKGSQVLLSRYGLGRNPKVWADPLCFKPERHLNECSEVTLTENDLRFISFSTGKRGCAAPALGTALTTMMLARLLQGFTWKLPENETRVELMESSHDMFLAKPLVMVGDLRLPEHLYPTVK* |
| *Sb*CYP79A1 558\|521 amino acids [native\|ΔTM] |
| MATMEVEAAAATVLAAPLLSSSAILKLLLFVVTLSYLARALRRPRKSTTKCSSTTCASPPAGVGNPPLPPGPVPWPVVGNLPEMLLNKPAFRWIHQMMREMGTDIACVKLGGVHVVSITCPEIAREVLRKQDANFISRPLTFASETFSGGYRNAVLSPYGDQWKKMRRVLTSEIICPSRHAWLHDKRTDEADNLTRYVYNLATKAATGDVAVDVRHVARHYCGNVIRRLMFNRRYFGEPQADGGPGPMEVLHMDAVFTSLGLLYAFCVSDYLPWLRGLDLDGHEKIVKEANVAVNRLHDTVIDDRWRQWKSGERQEMEDFLDVLITLKDAQGNPLLTIEEVKAQSQDITFAAVDNPSNAVEWALAEMVNNPEVMAKAMEELDRVVGRERLVQESDIPKLNYVKACIREAFRLHPVAPFNVPHVALADTTIAGYRVPKGSHVILSRTGLGRNPRVWDEPLRFYPDRHLATAASDVALTENDLRFISFSTGRRGCIAASLGTAMSVMLFGRLLQGFTWSKPAGVEAVDLSESKSDTFMATPLVLHAEPRLPAHLYPSISI* |
| *Bv*CYP79F6 P-type 536\|508 amino acids [native\|ΔTM] |
| MMSVTTSLPYPFQILLVFIISTASITLLGRMLSRPTKTKDRSRQLPPGPRGWPILGNLPELIMVRPKYKYFHNAMKQLNTEIACFNFAGTHAITINSDEIAREAFKERDADFASRPDVFSMRTIGDNCKSMGNSPYGEQFVKMKRVITTELMSAKTLNMMVASRTIEADNLIAYVHSMYQRSETVDLRELSRVYGYAMTMRMMFGRRNITKDNVLSEDGRLTKAERGHLDAIFDTLNCLPSFSPADYLERWFKGWNIDGQEEMVKKSCNFVRSYNNPIIDERVKLWREKGGKAAVEDWIDTFITLKDENGKYLITPDEIKAQCVEFCIAAIDNPANNMEWTLAEMLKNPEVLRKALKELDEVVGKERLVQESDIPNLNYIKACCRETFRIHPSAHFIPPHVARQDTTLGGYFIPKGSHIHICRSELGRSPKIWKDPMVYKPERHLEDGISKEVTLVETELRFVSFGTGRRGCVGVKVGTIMMVTLLARFLQAFNWKLDPGFGPLSLEEDDVMLMAKPLLLSVEPRLAPNLYPKFRH* |
| *Me*CYP79D2 541\|501 amino acids [native\|ΔTM] |
| MAMNVSTTATTTASFASTSSMNNTAKILLITLFISIVSTVIKLQKRASYKKASKNFPLPPGPTPWPLIGNIPEMIRYRPTFRWIHQLMKDMNTDICLIRFGKTNVVPISCPVIAREILKKHDAVFSNRPKILCAKTMSGGYLTTIVVPYNDQWKKMRKVLTSEIISPARHKWLHDKRAEEADQLVFYINNQYKSNKNVNVRIAARHYGGNVIRKMMFSKRYFGKGMPDGGPGPEEIMHVDAIFTALKYLYGFCISDYLPFLEGLDLDGQEKIVLNANKTIRDLQNPLIEERIQQWRSGERKEMEDLLDVFITLQDSDGKPLLNPDEIKNQIAEIMIATIDNPANAVEWAMGELINQPELLAKATEELDRVVGKDRLVQESDIPNLNYVKACAREAFRLHPVAYFNVPHVAMEDAVIGDYFIPKGSWAILSRYGLGRNPKTWPDPLKYDPERHLNEGEVVLTEHDLRFVTFSTGRRGCVAALLGTTMITMMLARMLQCFTWTPPPNVTRIDLSENIDELTPATPITGFAKPRLAPHLYPTSP* |

Table S6. Transitions and response factors used for selected reaction monitoring during analysis of phenylacetaldoxime (Phe-Ox) and Phe-Ox glutathione conjugate (Phe-GSH) by LC-MS/MS. *(E)-* and *(Z)-* form of Phe-Ox were detected separately but combined for calculations. Retention times and precursor ion to fragment ion transitions are provided. Q denotes the quantifier ion transition. Other transitions were used for metabolite identification.

| **Analyte** | **RT [min]** | **Q1 [M+H]^+^** | **Q3 [M+H] ^+^** | **CE [eV]** | **Internal standard** | **Q1 [M+H] ^+^** | **Q3 [M+H] ^+^** | **CE [eV]** | **Response factor** |
| --- | --- | --- | --- | --- | --- | --- | --- | --- | --- |
| Phe-Ox (*E*) | 4.14 | 136.1 | 118.1^Q^ | -10 | 13C,15N-Phe | 176.2 | 129.2 | -14 | 6.98 |
|  |  | 136.1 | 58.3 | -12 |  |  |  |  |  |
|  |  | 136.1 | 91.1 | -30 |  |  |  |  |  |
| Phe-Ox (*Z*) | 4.34 | 136.1 | 118.1^Q^ | -10 | 13C,15N-Phe | 176.2 | 129.2 | -14 | 6.98 |
|  |  | 136.1 | 58.3 | -12 |  |  |  |  |  |
|  |  | 136.1 | 91.1 | -30 |  |  |  |  |  |
| Phe-GSH | 3.27 | 441.1 | 279.1^Q^ | -15 | 13C,15N-Phe | 176.2 | 129.2 | -14 | 7.19 |
|  |  | 441.1 | 131 | -36 |  |  |  |  |  |
|  |  | 441.1 | 311 | -8 |  |  |  |  |  |

Table S7. Transitions used for selected reaction monitoring for targeted proteomics on peptides of CYP79A2 and isocitrate dehydrogenase (ICD). Retention times and precursor ion to fragment ion transitions are provided. All peptides were selected in Q1 as the double-charged ions [M+H]2+ and fragment ions in Q3 as single-charged ions [M+H]+. Collision energies (CE) for natural non-labelled (light) and isotopically labelled (heavy) peptides were identical.

|  |  |  | Light peptide | | Heavy peptide | |  |  |
| --- | --- | --- | --- | --- | --- | --- | --- | --- |
| **Protein** | **Peptide** | **RT [min]** | **Q1**  **[M+H]^2+^** | **Q3**  **[M+H]^+^** | **Q1**  **[M+H]^2+^** | **Q3**  **[M+H]^+^** | **Fragmentor voltage [V]** | **CE**  **[eV]** |
| CYP79A2 | SWPLIGNLPEILGR^a^ | 21.60 | 782.94 | 1081.64 | 787.95 | 1091.64 | 130 | -17 |
| (At5g05260) |  | 21.60 | 782.94 | 968.55 | 787.95 | 978.56 | 130 | -33 |
|  |  | 21.60 | 782.94 | 684.40 | 787.95 | 694.41 | 130 | -29 |
|  | GNAFVVIDLR^a^ | 8.80 | 552.31 | 861.52 | 557.32 | 871.53 | 130 | -18 |
|  |  | 8.80 | 552.31 | 714.45 | 557.32 | 724.46 | 130 | -18 |
|  |  | 8.80 | 552.31 | 615.38 | 557.32 | 625.39 | 130 | -10 |
|  | LVIESDLPNLNYVK*^a^ | 15.30 | 808.94 | 1291.65 | 812.95 | 1299.67 | 130 | -18 |
|  |  | 15.30 | 808.94 | 1162.61 | 812.95 | 1170.62 | 130 | -30 |
|  |  | 15.30 | 808.94 | 847.47 | 812.95 | 855.48 | 130 | -26 |
|  | LIQGFTWLPVPGK^a^ | 18.40 | 728.42 | 1101.61 | 732.42 | 1109.62 | 130 | -19 |
|  |  | 18.40 | 728.42 | 497.31 | 732.42 | 505.32 | 130 | -27 |
|  |  | 18.40 | 728.42 | 301.19 | 732.42 | 309.20 | 130 | -31 |
|  | YNDPFVDER^b^ | 8.10 | 577.76 | 877.41 | 582.76 | 887.41 | 130 | -14 |
|  |  | 8.10 | 577.76 | 762.38 | 582.76 | 772.39 | 130 | -18 |
|  |  | 8.10 | 577.76 | 665.33 | 582.76 | 675.33 | 130 | -22 |
|  | FLDLEGHEK^b^ | 7.50 | 544.27 | 827.39 | 548.28 | 835.40 | 130 | -17 |
|  |  | 7.50 | 544.27 | 712.36 | 548.28 | 720.38 | 130 | -17 |
|  |  | 7.50 | 544.27 | 599.28 | 548.28 | 607.29 | 130 | -17 |
| ICD | GPLTTPVGGGIR*^a^ | 8.80 | 562.83 | 857.49 | 567.83 | 867.49 | 130 | -20 |
|  |  | 8.80 | 562.83 | 756.44 | 567.83 | 766.44 | 130 | -21 |
|  |  | 8.80 | 562.83 | 655.39 | 567.83 | 665.40 | 130 | -19 |
|  | TVTYDFER^b^ | 7.10 | 515.75 | 830.37 | 525.75 | 840.38 | 130 | -13 |
|  |  | 7.10 | 515.75 | 729.32 | 525.75 | 739.33 | 130 | -17 |
|  |  | 7.10 | 515.75 | 566.26 | 525.75 | 576.27 | 130 | -17 |

*= Peptides represented in Figure 2C; a= Peptide previously reported by Petersen et al. 2019; b= this study

Table S8. List of all plasmids created and used during this study.

| **#** | **Plasmid** | **Description** | **Reference** |
| --- | --- | --- | --- |
| 1 | pCDF1b | used to construct exp. vectors | Novagen |
| 2 | pET52b(+) | used to construct exp. vectors | Novagen |
| 3 | pRI952 | carries ileX and argU | (1) |
| 4 | pET52-CYP79A2-ATR1 | exp. of CYP79A2-ATR1 operon | This study |
| 5 | pET52-Δ14CYP79A2-ATR1 | exp. of [ΔTM]CYP79A2-ATR1 operon | This study |
| 6 | pET52-Δ23CYP79A2-ATR1 | exp. of [ΔTM+]CYP79A2-ATR1 operon | This study |
| 7 | pET52-bCYP79A2-ATR1 | exp. of [÷Barnes]CYP79A2-ATR1 operon | This study |
| 8 | pET52-Barnes-CYP79A2-ATR1 | exp. of [+Barnes]CYP79A2-ATR1 operon | This study |
| 9 | pET52-[OmpA]CYP79A2-ATR1 | exp. of [OmpA]CYP79A2-ATR1 operon | This study |
| 10 | pET52-[SohB]Δ14CYP79A2-ATR1 | exp. of [SohB]CYP79A2-ATR1 operon | This study |
| 11 | pET52-[28aa]CYP79A2-ATR1 | exp. of [28aa]CYP79A2-ATR1 operon | This study |
| 12 | pET52-CYP79A2-CYP83B1-ATR1 | exp. of CYP79A2-CYP83B1-ATR1 operon | This study |
| 13 | pET52-Δ14CYP79A2-CYP83B1-ATR1 | exp. of [ΔTM]CYP79A2-CYP83B1-ATR1 operon | This study |
| 14 | pET52-Δ14CYP79A2-Δ23CYP83B1-ATR1 | exp. of [ΔTM]CYP79A2-[ΔTM]CYP83B1-ATR1 operon | This study |
| 15 | pET52-Δ14CYP79A2-Δ28CYP83B1-ATR1 | exp. of [ΔTM]CYP79A2-[ΔTM+]CYP83B1-ATR1 operon | This study |
| 16 | pET52-Δ14CYP79A2-bCYP83B1-ATR1 | exp. of [ΔTM]CYP79A2-[÷Barnes]CYP83B1-ATR1 operon | This study |
| 17 | pET52-Δ14CYP79A2-[Barnes]CYP83B1-ATR1 | exp. of [ΔTM]CYP79A2-[+Barnes]CYP83B1-ATR1 operon | This study |
| 18 | pET52-Δ14CYP79A2-[OmpA]CYP83B1-ATR1 | exp. of [ΔTM]CYP79A2-[OmpA]CYP83B1-ATR1 operon | This study |
| 19 | pET52-Δ14CYP79A2-[SohB]Δ23CYP83B1-ATR1 | exp. of [ΔTM]CYP79A2-[SohB]CYP83B1-ATR1 operon | This study |
| 20 | pET52-Δ14CYP79A2-[28aa]CYP83B1-ATR1 | exp. of [ΔTM]CYP79A2-[28aa]CYP83B1-ATR1 operon | This study |
| 21 | pET52-CYP79A2-CYP83A1-ATR1 | exp. of CYP79A2-CYP83A1-ATR1 operon | This study |
| 22 | pET52-Δ14CYP79A2-CYP83A1-ATR1 | exp. of [ΔTM]CYP79A2-CYP83A1-ATR1 operon | This study |
| 23 | pET52-Δ14CYP79A2-Δ21CYP83A1-ATR1 | exp. of [ΔTM]CYP79A2-[ΔTM]CYP83A1-ATR1 operon | This study |
| 24 | pET52-Δ14CYP79A2-Δ29CYP83A1-ATR1 | exp. of [ΔTM]CYP79A2-[ΔTM+]CYP83A1-ATR1 operon | This study |
| 25 | pET52-Δ14CYP79A2-bCYP83A1-ATR1 | exp. of [ΔTM]CYP79A2-[÷Barnes]CYP83A1-ATR1 operon | This study |
| 26 | pET52-Δ14CYP79A2-[Barnes]CYP83A1-ATR1 | exp. of [ΔTM]CYP79A2-[+Barnes]CYP83A1-ATR1 operon | This study |
| 27 | pET52-Δ14CYP79A2-[OmpA]CYP83A1-ATR1 | exp. of [ΔTM]CYP79A2-[OmpA]CYP83A1-ATR1 operon | This study |
| 28 | pET52-Δ14CYP79A2-[SohB]Δ21CYP83A1-ATR1 | exp. of [ΔTM]CYP79A2-[SohB]CYP83A1-ATR1 operon | This study |
| 29 | pET52-Δ14CYP79A2-[28aa]CYP83A1-ATR1 | exp. of [ΔTM]CYP79A2-[28aa]CYP83A1-ATR1 operon | This study |
| 30 | pET52-[SohB]Δ14CYP79A2-CYP83A1-ATR1 | exp. of [SohB]CYP79A2-CYP83B1-ATR1 operon | This study |
| 31 | pCDF-GSTF11 | exp. of GSTF11 | This study |
| 32 | pCDF-GSTF9 | exp. of GSTF9 | This study |
| 33 | pET52-CYP79A1-CYP83B1-ATR1 | exp. of CYP79A1-CYP83B1-ATR1 operon | This study |
| 34 | pET52-CYP79B2-CYP83B1-ATR1 | exp. of CYP79B2-CYP83B1-ATR1 operon | This study |
| 35 | pET52-CYP79D2-CYP83B1-ATR1 | exp. of CYP79D2-CYP83B1-ATR1 operon | This study |
| 36 | pET52-CYP79F6-CYP83A1-ATR1 | exp. of CYP79F6-CYP83A1-ATR1 operon | This study |
| 37 | pET52-Δ38CYP79A1-Δ23CYP83B1-ATR1 | exp. of [ΔTM]CYP79A1-[ΔTM]CYP83B1-ATR1 operon | This study |
| 38 | pET52-Δ42CYP79B2-Δ23CYP83B1-ATR1 | exp. of [ΔTM]CYP79B2-[ΔTM]CYP83B1-ATR1 operon | This study |
| 39 | pET52-Δ41CYP79D2-Δ23CYP83B1-ATR1 | exp. of [ΔTM]CYP79D2-[ΔTM]CYP83B1-ATR1 operon | This study |
| 40 | pET52-Δ29CYP79F6-Δ21CYP83A1-ATR1 | exp. of [ΔTM]CYP79F6-[ΔTM]CYP83A1-ATR1 operon | This study |
| 41 | pCDF-ATR1 | exp. of ATR1 | This study |
| 42 | pCDF-ATR2 | exp. of ATR2 | This study |
| 43 | pCDF-Δ44ATR2 | exp. of Δ44ATR2 | This study |

exp. = expression

**Supplementary Material References**

1. Del Tito BJ, Ward JM, Hodgson J, Gershater CJ, Edwards H, Wysocki LA, et al. Effects of a minor isoleucyl tRNA on heterologous protein translation in *Escherichia coli*. J Bacteriol. 1995;177:7086–91. <https://doi.org/10.1128/jb.177.24.7086-7091.1995>

2. Jander G, Norris SR, Joshi V, Fraga M, Rugg A, Yu S, et al. Application of a high-throughput HPLC-MS/MS assay to *Arabidopsis* mutant screening; evidence that threonine aldolase plays a role in seed nutritional quality. Plant J. 2004;39:465–75. <https://doi.org/10.1111/j.1365-313x.2004.02140.x>

3. Docimo T, Reichelt M, Schneider B, Kai M, Kunert G, Gershenzon J, et al. The first step in the biosynthesis of cocaine in *Erythroxylum coca*: the characterization of arginine and ornithine decarboxylases. Plant Mol Biol. 2012;78:599–615. <https://doi.org/10.1007/s11103-012-9886-1>

4. Petersen A, Crocoll C, Halkier BA. De novo production of benzyl glucosinolate in *Escherichia coli*. Metab Eng. 2019;54:24–34. <https://doi.org/10.1016/j.ymben.2019.02.004>

5. Crocoll C, Halkier BA, Burow M. Analysis and Quantification of Glucosinolates. Curr Protoc Plant Biol. 2016;1:385–409. <https://doi.org/10.1002/cppb.20027>

6. Percy AJ, Chambers AG, Yang J, Domanski D, Borchers CH. Comparison of standard- and nano-flow liquid chromatography platforms for MRM-based quantitation of putative plasma biomarker proteins. Anal Bioanal Chem. 2012;404:1089–101. <https://doi.org/10.1007/s00216-012-6010-y>

7. Batth TS, Keasling JD, Petzold CJ. Targeted proteomics for metabolic pathway optimization. Methods Mol Biol. 2012;944:237–49. <https://doi.org/10.1007/978-1-62703-122-6_17>

8. Geu-Flores F, Møldrup ME, Böttcher C, Olsen CE, Scheel D, Halkier BA. Cytosolic γ-Glutamyl Peptidases Process Glutathione Conjugates in the Biosynthesis of Glucosinolates and Camalexin in *Arabidopsis*. Plant Cell. 2011;23:2456–69. <https://doi.org/10.1105/tpc.111.083998>

9. Xie C, Zhong D, Chen X. A fragmentation-based method for the differentiation of glutathione conjugates by high-resolution mass spectrometry with electrospray ionization. Anal Chim Acta. 2013;788:89–98. <https://doi.org/10.1016/j.aca.2013.06.022>
